# Theoretical Study on the Biosynthesis of the Mandapamates: Mechanistic Insights Using Density Functional Theory

**DOI:** 10.1021/acs.joc.4c00859

**Published:** 2024-09-09

**Authors:** Di Wang, Gerald Pattenden, Kam Loon Fow, Michael J. Stocks, Jonathan D. Hirst, Bencan Tang

**Affiliations:** † Department of Chemical and Environmental Engineering, Nottingham Ningbo China Beacons of Excellence Research and Innovation Institute, Key Laboratory for Carbonaceous Waste Processing and Process Intensification Research of Zhejiang Province, the University of Nottingham Ningbo China, 199 Taikang East Road, Ningbo 315100, P. R. China; ‡ School of Chemistry, University of Nottingham, University Park, Nottingham NG7 2RD, U.K.; § Nottingham University Biodiscovery Institute, School of Pharmacy, University of Nottingham, University Park, Nottingham NG7 2RD, U.K.

## Abstract

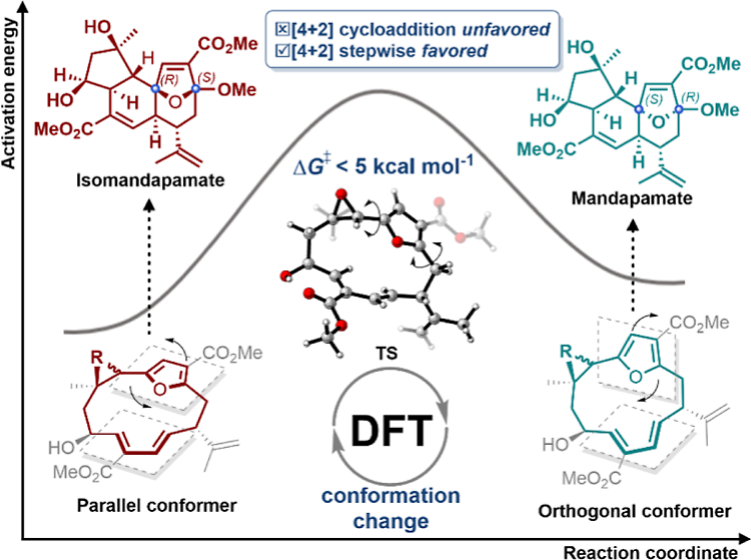

Density functional theory (B3LYP-D3(BJ) and ωB97XD) calculations have been used to assess the stereochemical outcomes of the proposed transannular [4 + 2] cycloaddition pathway for the biosynthesis of mandapamate and isomandapamate from macrocyclic intermediates. Calculations reveal that the topological shift between macrocyclic conformers is vital in controlling the stereoselectivity of the downstream steps toward the isomeric mandapamates. A stepwise 4 + 2 type process is energetically favored over a concerted [4 + 2] pathway at room temperature, and is consistent with the stereochemistries found in the natural products.

## Introduction

**Figure 1:**
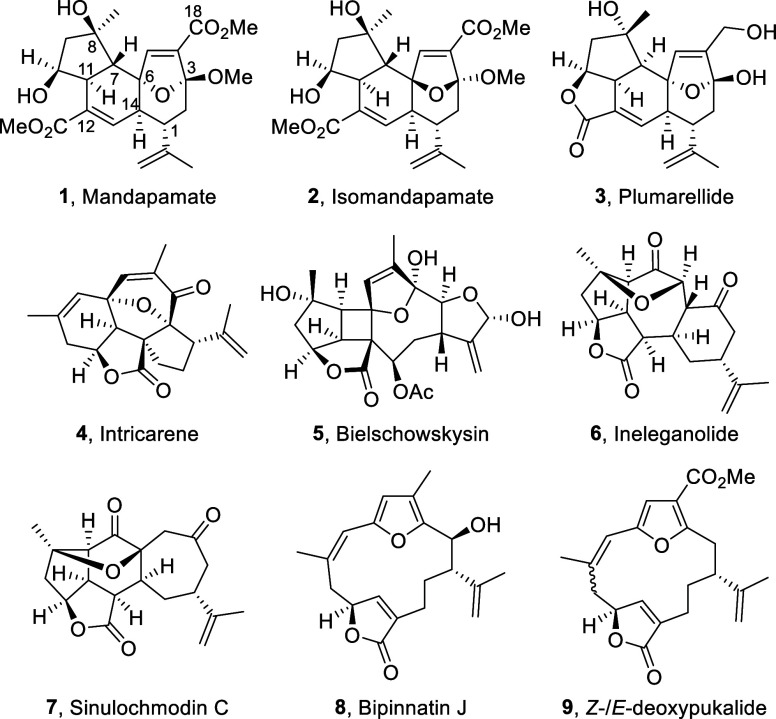
Representative polycyclic cembranoid diterpenes, C19 norcembranoids, and macrocyclic furanocembranes.

**Scheme 1:**
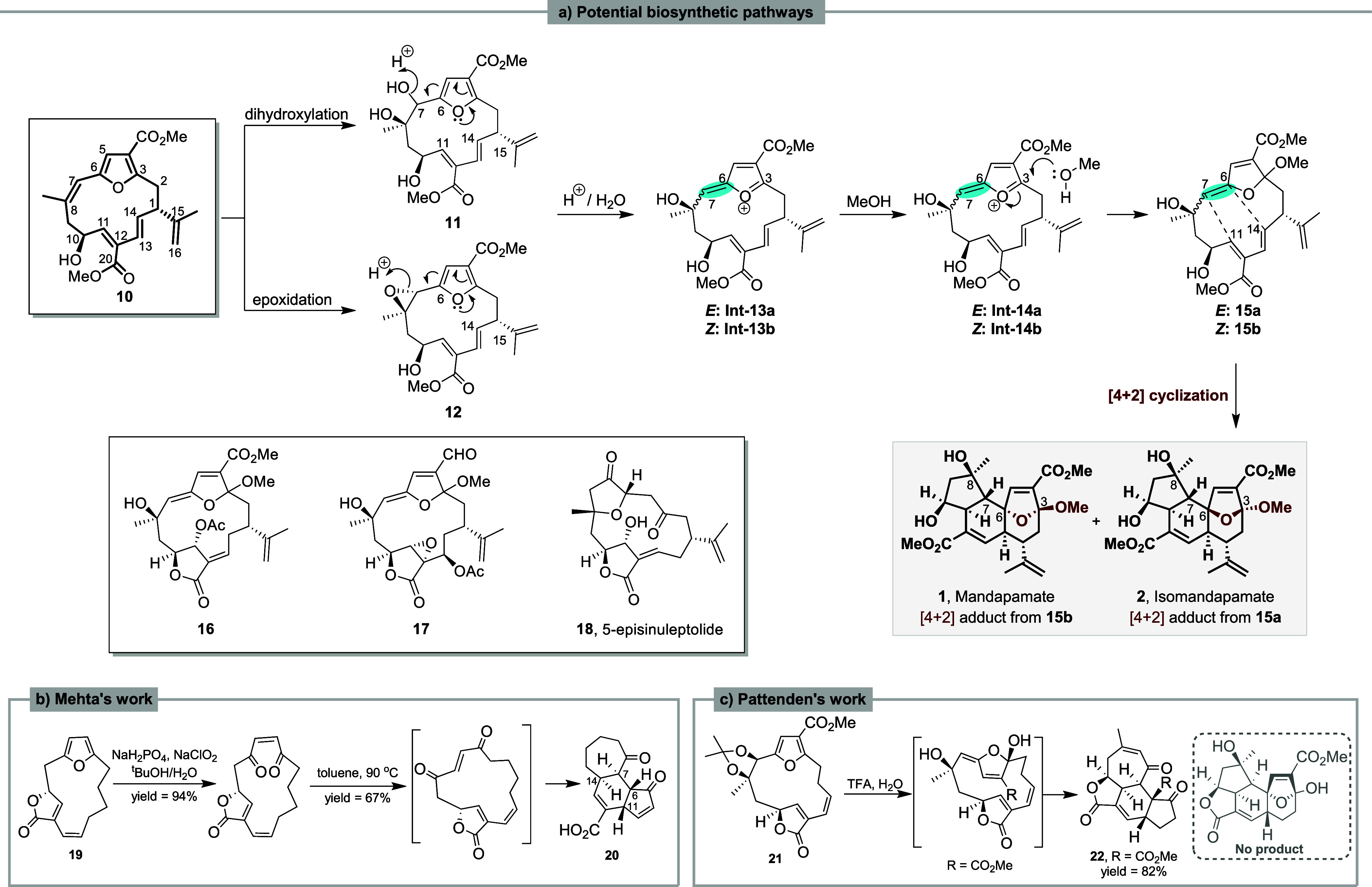
(a) Biosynthetic Proposal for the Formation of Mandapamate 1 and Isomandapamate 2 (b and c) Reported Synthetic Attempts towards Mandapamate **1** and Isomandapamate **2** Ring System.

Mandapamate **1** and its stereoisomer isomandapamate **2** are novel polycyclic cembranoid diterpenes found in soft corals.^1^ Other polycyclic cembranoids found in soft corals include plumarellide **3**, intricarene **4** and bielschowskysin **5**, and the closely related C19 norditerpenes ineleganolide **6** and sinulochmodin C (**7**).^2^ Each of these polycyclic structures is purported to be derived in nature from macrocyclic furanocembranoid structures, e.g., **8**(3) and **9**,^4^ via sequences involving oxidation/hydration followed by transannular cyclizations (Figure 1).^5^ Thus, in the case of the mandapamates **1** and **2**, they are thought to be biosynthesised from the hypothetical precursor **10** via **11**/**12**, leading to the key *exo*-enol ether intermediate **15** which then undergoes transannular [4 + 2] cycloaddition (Scheme 1a). Stable *exo*-enol ether structures similar to **15** were first reported in corals by Fenical et al.^6^ Others were described later, e.g., **16** and **17**,^7^ and *exo* enol ether structures are also thought to be implicated in biosynthetic pathways which lead to plumarellide **3** and to bielschowskysin **5**.^5^

Mandapamate **1** and isomandapamate **2** share a common angular [5,6,7]-carbocyclic ring system with (3*R*, 6*S*)- and (3*S*, 6*R*)-stereochemistry respectively at their C3 and C6 centers (Figure 1). This stereogenicity originates from the relative orientation of the oxygen atoms on the bridged cycloheptenes in their structures. We postulated that during the proposed biosynthesis of the mandapamates there is likely to be restraints imposed on the conformations of their macrocyclic enol ether/furanoxonium ion intermediates, *viz*. **13**–**15** (Scheme 1a), which would predetermine the stereochemical outcomes of the subsequent transannular cyclizations, thereby leading to the different stereochemistries present in the two natural products. The origins of these proposed conformational preferences are not known, which has motivated us to establish some theoretical support for their existence, and thereby importance, using density functional theory (DFT).

The complexities of the ring systems and the oxidation patterns present in the naturally occurring polycycles **1**–**7**, combined with the interesting biological properties they display, have made them challenging targets for the synthetic chemist. Pertinent to our study, there has been a recent novel synthesis of the core carbocyclic ring system in bielschowskysin **5** which was based on an intramolecular [2 + 2] cyclization involving an *exo* enol ether intermediate akin to that shown in compound **15**.^8,9^ Other synthetic studies implicating enol ether intermediates have been carried out toward plumarellide **3**,^10^ and total syntheses of both ineleganolide **6** and sinulochmodin C (**7**) have recently been accomplished.^11^ Earlier Pattenden et al.^12^ and Trauner et al.^13^ independently achieved a biomimetic synthesis of (+)-intricarene **4** from bipinnatin J (**8**) implicating an intramolecular dipolar [5 + 2] cycloaddition reaction as the key step, and in 2011, Li and Pattenden presented biomimetic syntheses of ineleganolide **6** and sinulochmodin C (**7**) from the macrocyclic 5-episinuleptolide **18** (believed to be derived from deoxypukalide **9** in vivo) via sequences of transannular Michael reactions.^14^ Some studies have been made toward the synthesis of the mandapamates **1** and **2** exploring an intramolecular [4 + 2] cyclization strategy to produce their [5,6,7] tricyclic ring systems, *viz*. **19** to **20** and **21** to **22** (Scheme 1b, c),^15,16^ but with limited success.

The challenges presented by the synthesis of the naturally occurring polycycles **1**–**7**, implicating their likely biosynthesis precursors, have also prompted theoretical chemists to examine features of the mechanisms of these likely transformations. Quantum chemical calculations are now widely used to uncover new mechanisms^17^ as well as seek theoretical support for biosynthetic proposals.^18^ Related computational studies have recently been reviewed.^18a^ Tang and Paton, for example, recently used DFT to probe the pathway to the cyclobutane rings in the furanocembranoid providencin^19^ and in bielschowskysin **5**. Thus, based on quantum chemical calculations of the thermal and photochemical pathways toward the formation of the cyclobutane ring in bielschowskysin **5**, they concluded that while a photo [2 + 2] cycloaddition pathway is computed to be highly efficient, a thermal ring-closure process in a single step in water is also energetically feasible at room temperature.^20^ More recently, alongside their synthetic work toward bielschowskysin **5**, Scesa et al. also used DFT calculations to analyze the conformation of the likely furanocembranoid precursors, e.g., deoxypukalide **9** to its formation.^8b^ Earlier, Lygo et al. used DFT calculations to study the steps leading to the polycyclic ring system found in plumarellide **3** and compared a stepwise cyclization pathway with a [4 + 2] cycloaddition of a furanoxonium ion intermediate.^21^ Hence, transannular cycloaddition reactions implicating furanoxonium ions as key intermediates have been verified both experimentally^5b,5e,10,16^ and computationally with DFT.^22^

In the proposed biosynthesis of the mandapamates **1** and **2** from the furanocembranoid precursor **10** (Scheme 1a) the key *exo* enol ether intermediate **15**, can exhibit both *E*- and *Z*-geometry, each of which can undergo either a concerted or stepwise transannular 4 + 2 cycloaddition process leading to the isomeric mandapamates. Known furancembranes akin to the hypothetical precursor **10** have been isolated and/or synthesized, including bipinnatin J (**8**),^3,12,13^*Z*-/*E*-deoxypukalide **9**,^4^ leptolide,^23^ pukalide aldehyde,^23^ molestin E,^24^ in addition to two model precursors designed by Mehta^15^ and Pattenden.^16^ The existence of these furancembranes gives us confidence that our proposed precursors could be derived from naturally existing furancembranes, if they themselves are not natural products.

To gain insight into this biosynthesis proposal we have carried out detailed quantum chemical calculations of the key steps and the likely stereochemical and conformational requirements of any macrocyclic furanoxonium ion intermediate implicated in competitive transannular 4 + 2 cycloaddition processes. The key point for this calculation is to understand the feasibility of the proposed transannular reactions leading to mandapamate and isomandapamate, despite the possibility that some side reactions might occur experimentally. For example, transannular cycloaddition between the furan ring and the *exo* C(15)=C is a possibility. However, our calculations revealed that the associated transition state (TS) has a very high activation energy barrier of over 59.0 kcal mol^–1^, which rules out this pathway (Figure S7). Similarly, selective dihydroxylation or epoxidation of the C7–C8 double bond with the existence of C15–C16 double bond could be problematic. Therefore, a good number of synthetic attempts/trails would be required to find the right reaction conditions to achieve these transformations.

## Computational Methods

All the calculations reported were carried out in Gaussian 16.^25^ Unless stated otherwise, B3LYP-D3(BJ)^26^/def2-TZVP^27^//B3LYP/6-31G(d)^28^ and ωB97XD^29^/def2-TZVP//B3LYP/6-31G(d) relative free energies at 298.15 K, obtained from optimized species including an implicit SMD description of water,^30^ are quoted throughout, the latter in parentheses. The vibrational frequencies were computed at the same level of theory as for the geometry optimizations to confirm whether each optimized structure is an energy minimum (possessing zero imaginary frequencies) or a TS (possessing one single imaginary frequency), and to obtain the zero-point vibrational energy and thermal corrections under 298.15 K and 1 atm pressure. All transition states were confirmed to connect reactants and products by intrinsic reaction coordinate calculations.^31^ All Gibbs free energies discussed in the manuscript were corrected using the quasi-rigid rotor-harmonic oscillator approach proposed by Grimme^32^ and implemented in the GoodVibes code^33^ (Figures S5, S6 and Table S5). The 3D graphics of molecules were generated using CYLview.^34^ Electrostatic potential surface (EPS) analysis based on the noncovalent interactions was performed using Multiwfn (v3.8_dev)^35^ and visualized using VMD.^36^ The level of the theory that we have adopted was based on functional/basis set benchmark studies carried out in this work (Tables S1, S2 and S3). The B3LYP-D3(BJ) and ωB97XD functionals provided similar accuracy and qualitative conclusions. We selected B3LYP/6-31G(d) level for the geometry optimization in order to achieve the best balance between accuracy and computational cost. The 0.0 kcal mol^–1^ reference includes the single point energies of **11b**, H_2_O, H_3_O^+^ and CH_3_OH. The structures of H_2_O, H_3_O^+^ and CH_3_OH were optimized and computed at the same level of **11b**. Conformational analyses of **11** and **12** were carried out via random searching in the GMMX 3.1 module of Gaussian 16 using the MMFF94^37^ force field. The computational details are given in the Supporting Information.

## Results and Discussion

### Conformational Analysis of the Macrocyclic Furanocembranes 11/12 to 13

**Figure 2:**
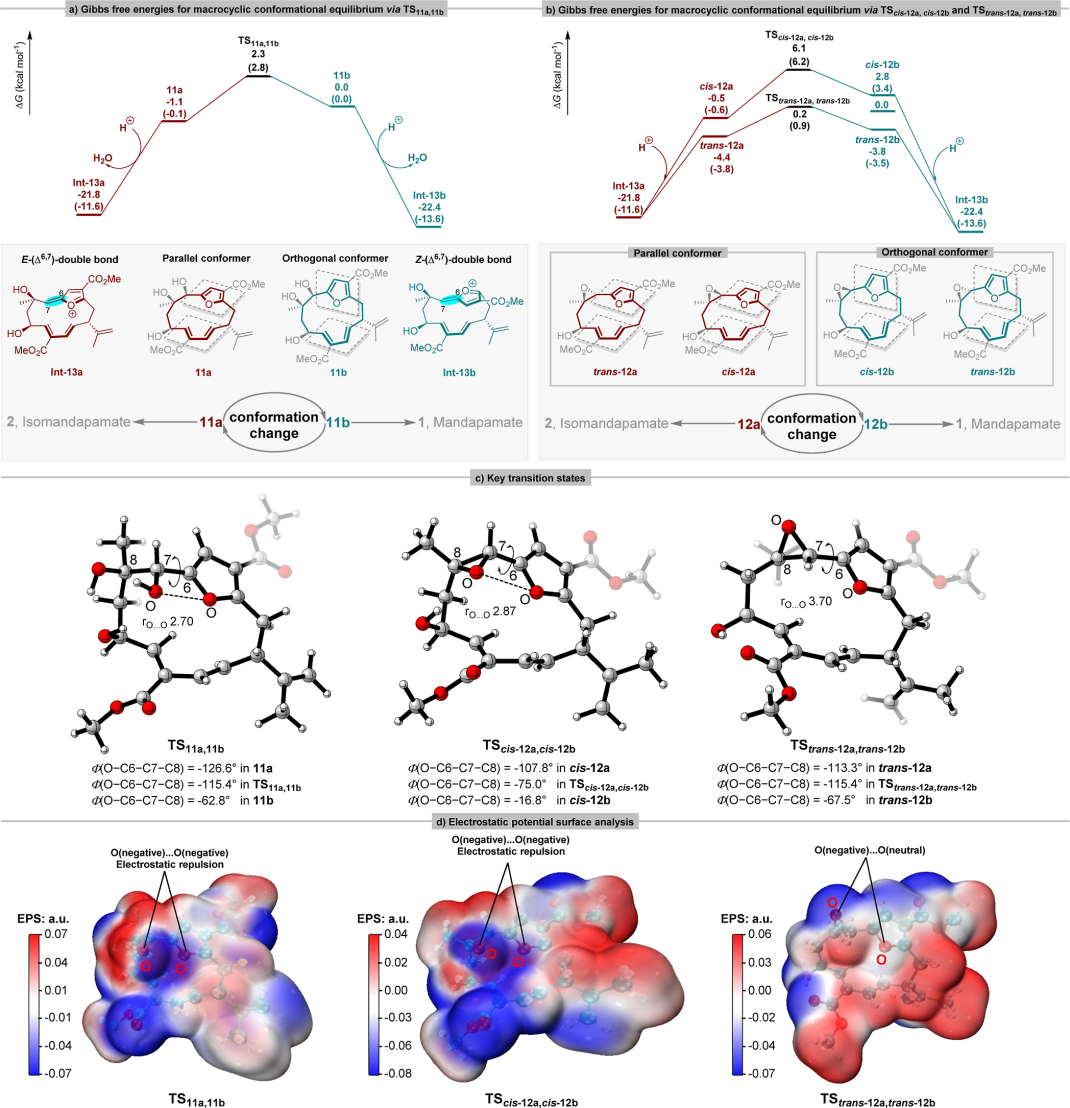
(a, b) Calculated conformational energy profiles and transformations toward **Int-13a** and **Int-13b** respectively. Free energies calculated with SMD(H_2_O)-B3LYP-D3(BJ)/def2-TZVP//B3LYP/6-31G(d) and SMD(H_2_O)-ωB97XD/def2-TZVP//B3LYP/6-31G(d) (quoted in parentheses) respectively. All energies are relative to that of **11b**. (c) Color scheme of key transition states: H, white; C, gray; O, red. Bond distances in Å. (d) EPS analysis of the transition states **TS**_**11a,11b**_, **TS**_*cis***-12a,***cis***-12b**_ and **TS**_*trans***-12a,***trans***-12b**_.

Referring to the biosynthesis proposal (Scheme 1a) we first analyzed the expected topological shift in the macrocyclic ring of the intermediates **11** to **13** resulting from conformational changes in the orientation of their furan rings. Using relaxed coordinate scans, the potential energy surfaces for these conformational changes were obtained at the B3LYP/6-31G(d) level through the incremental rotation of the O–C6–C7–C8 dihedral angles (10° increments each frame, see Supporting Information). As shown in Figure 2a, the energy minimum for conformer **11a** is almost equi-energetic with conformer **11b** (+1.1 kcal mol^–1^). These two conformers differ in the relative location of the furan-ring plane. The conformer **11a** is parallel while the conformer **11b** is orthogonal to the macrocycle. The activation Gibbs free energy toward the TS **TS**_**11a,11b**_ is 3.4 kcal mol^–1^ with a reverse barrier of 2.3 kcal mol^–1^ (**11b** → **TS**_**11a,11b**_), so that the interconversion between conformers **11a** and **11b** occurs readily. Following a pivotal exothermic process (furan dearomatisation), the conformers **11a** and **11b** are converted into the more stable furanoxonium ion intermediates **Int-13a** and **Int-13b** respectively. Interestingly, the configurations (*E*- vs *Z-*) of the Δ^6,7^ double bonds in **13** are divergent at this stage and they will be retained in the downstream steps, thereby controlling the C3 and C6 stereocenters in the final mandapamates. The pathways for the interconversion of the epoxy compounds (*cis***-12a**⇄*cis***-12b**, *trans***-12a**⇄*trans***-12b**) presented similar transition states **TS**_*cis***-12a,***cis***-12b**_ and **TS**_*trans***-12a,***trans***-12b**_ (Figure 2b). Compounds *cis***-12** and *trans***-12** differ in the orientations of their epoxy moieties. For the *cis* epoxide, the activation Gibbs free energy toward the TS **TS**_*cis***-12a,***cis***-12b**_ is 6.6 kcal mol^–1^ with a reverse barrier of 3.3 kcal mol^–1^ (*cis***-12b** → **TS**_*cis***-12a,***cis***-12b**_). In the corresponding *trans* epoxide, the activation Gibbs free energy toward the TS **TS**_*trans***-12a,***trans***-12b**_ is 4.6 kcal mol^–1^ with a reverse barrier of 4.0 kcal mol^–1^ (*trans***-12b** → **TS**_*trans***-12a,***trans***-12b**_). These two transition states (**TS**_*cis***-12a,***cis***-12b**_ and **TS**_*trans***-12a,***trans***-12b**_) also connect to the corresponding parallel conformers (*cis***-12a** and *trans***-12a**) and to the orthogonal conformers (*cis***-12b** and *trans***-12b**) which are in equilibrium and lead to the formation of the furanoxonium ion intermediates **Int-13a** and **Int-13b** with *E*- and *Z*-configured (Δ^6,7^)-double bonds, respectively. regioselectivity. To investigate further the origin of the conformational preference in the transition states **TS**_**11a,11b**_, **TS**_*cis***-12a,***cis***-12b**_ and **TS**_*trans***-12a,***trans***-12b**_, the EPS analysis was performed. As displayed in Figure 2c, d, in the transition states **TS**_**11a,11b**_ and **TS**_*cis***-12a,***cis***-12b**_, the moiety containing a hydroxy or epoxy group at C7 shows a negative surface potential (blue, *r*O···O 2.70 Å), while the oxygen atom on furan ring also shows a negative surface potential (blue, *r*O···O 2.87 Å), indicating that there is electrostatic repulsion between them. However, in the TS **TS**_*trans***-12a,***trans***-12b**_, the oxygen atom on the furan ring shows a neutral surface potential (white, *r*O···O 3.70 Å), indicating that there is less electrostatic interaction. This also explains why the activation energy barrier of **TS**_*cis***-12a,***cis***-12b**_ is higher than that of **TS**_*trans***-12a,***trans***-12b**_. Therefore, the EPS analysis suggests that the electrostatic interaction would be the driving force for the rotation about the furan ring.

### Pathway for the Formation of Mandapamate 1

**Figure 3:**
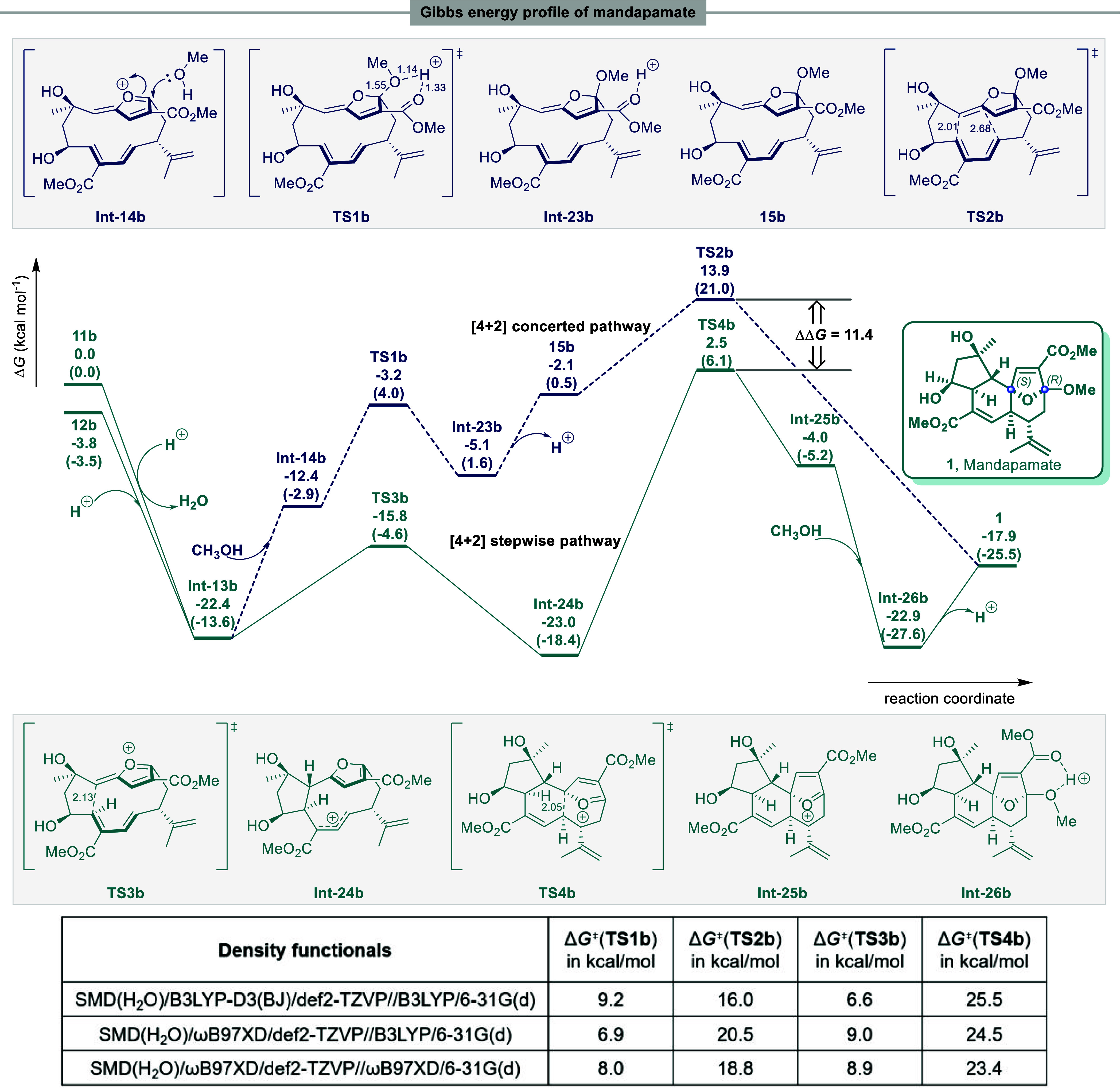
Calculated energy profile for the formation of mandapamate **1**. Geometry optimized with B3LYP/6-31G(d) in gas phase. Free energies calculated with SMD(H_2_O)-B3LYP-D3(BJ)/def2-TZVP//B3LYP/6-31G(d) and SMD(H_2_O)-ωB97XD/def2-TZVP//B3LYP/6-31G(d) (quoted in parentheses) respectively. Bond distances in Å.

As already discussed, the proposed biosynthesis of the mandapamates **1** and **2** (Scheme 1a) proceeds via the central stable furanoxonium ion intermediate **Int-13b** derived from **11b** and **12b**. The conformations of **11b** and **12b** are orthogonal and hence the intermediate **Int-13b** will have a *Z*-configured (Δ^6,7^)-double bond. This *Z*-configured (Δ^6,7^)-**Int-13b** is a direct optimization result of the pivotal furan dearomatisation process from **11b** and **12b**. Because extensive searches could not locate transition states connecting both sides of **11b**/**12b** and **int-13**, we consider that this furan dearomatisation process is exothermic and is likely to be a barrierless process on the PES. The furanoxonium ion intermediate **Int-13b** is next quenched by methanol leading to the intermediate **Int-14b** (Figure 3). This process proceeds via **TS1b** with an activation Gibbs free energy of 9.2 kcal mol^–1^. The resulting protonated intermediate **Int-23b** then undergoes deprotonation to afford the stable *exo* enol ether-cyclic ketal **15b**. Lastly, **15b** undergoes a transannular [4 + 2] cycloaddition via **TS2b** leading to mandapamate **1** with an activation Gibbs free energy of 16.0 kcal mol^–1^. However, the overall energy barrier of this pathway (**Int-13b** → **TS2b**, 36.3 kcal mol^–1^) seems to be overly high for a biosynthesis route. We therefore investigated the alternative, stepwise, intramolecular cyclization pathway from **13b** to mandapamate **1**. Indeed, our calculations demonstrated that the *Z*-configured (Δ^6,7^) intermediate **Int-13b** could undergo transannular C–C bond formation via **TS3b** (Figure 3) with an activation Gibbs free energy of 6.6 kcal mol^–1^ to generate the thermodynamically stable cyclopentane-substituted intermediate **Int-24b**. A second transannular C–C bond formation through **TS4b** then leads to the intermediate **Int-25b** containing the tricyclic ring system found in mandapamate **1** with an activation Gibbs free energy of 25.5 kcal mol^–1^. Methanolysis of the intermediate **Int-25b**, to **Int-26b**, followed by deprotonation finally produces mandapamate **1**. Attempts to locate the transition states connecting both sides of **int-25** and **int-26** (MeOH nucleophilic attack) were unsuccessful, indicating this is a barrierless process. Compared with the aforementioned concerted [4 + 2] cycloaddition pathway, the overall energy barrier in this stepwise route (**Int-13b** → **TS4b**, 24.9 kcal mol^–1^, without enzymatic intervention) is more reasonable. Our calculations revealed that B3LYP-D3(BJ) and ωB97XD functionals predicted the same rate-determining step, with M06-2X predicted a much lower Gibbs free activation energy for **TS4b**, see Table S3 in the Supporting Information, Δ*G*^⧧^(**TS4b**) is ca. 25 kcal mol^–1^ predicted by B3LYP-D3(BJ) and ωB97XD, but 21.4 kcal mol^–1^ predicted by M06-2X. One might anticipate that with enzymatic intervention, the energy barriers would typically decrease by at least a few kcal mol^–1^. The TS **TS3b** is favored over the **TS1b** by 12.6 kcal mol^–1^ and the TS **TS4b** is favored over the **TS2b** by 11.4 kcal mol^–1^ respectively.

### Pathway for the Formation of Isomandapamate 2

**Figure 4:**
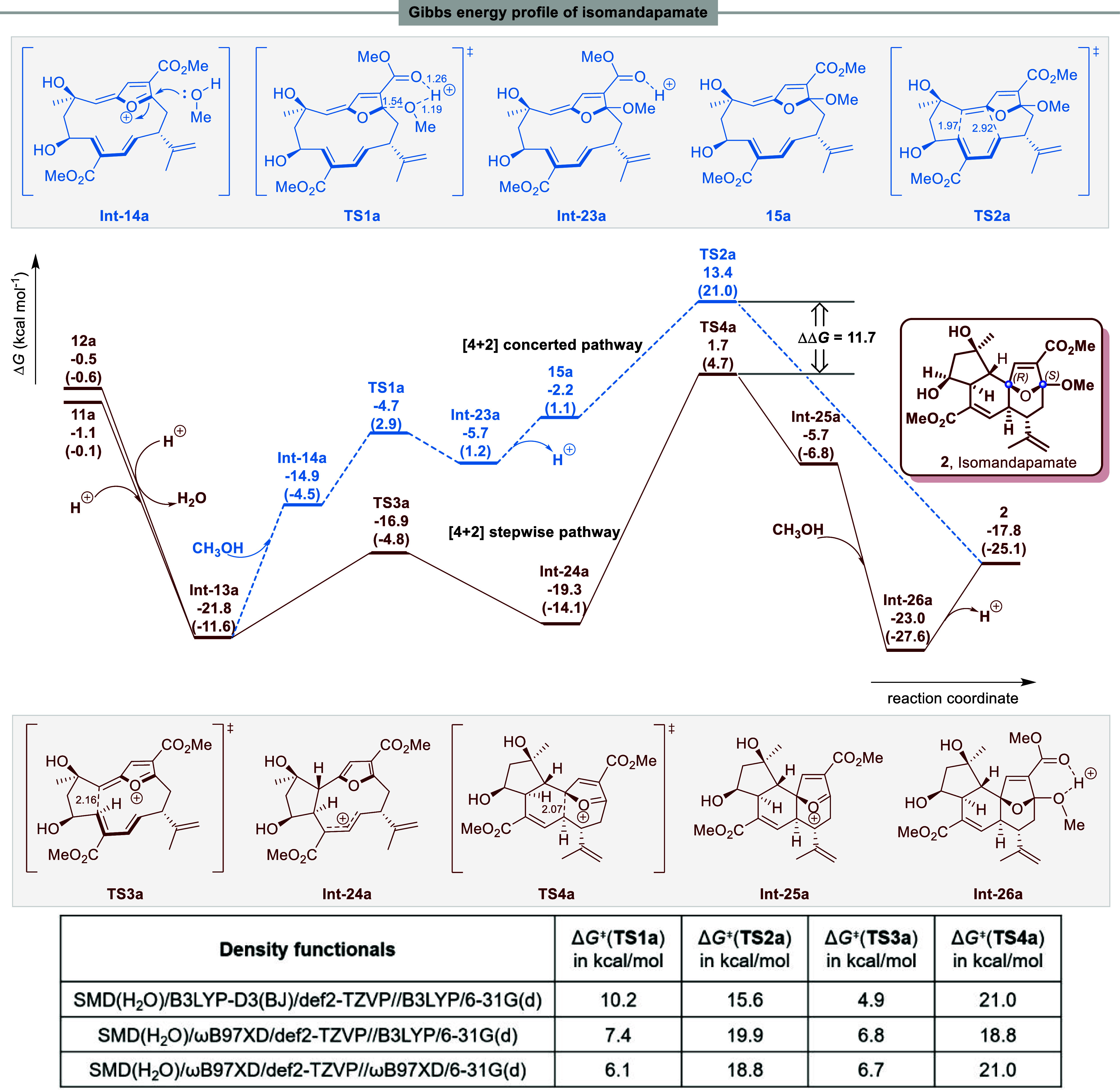
Calculated energy profile for the formation of isomandapamate **2**. Geometry optimized with B3LYP/6-31G(d) in gas phase. Free energies calculated with SMD(H_2_O)-B3LYP-D3(BJ)/def2-TZVP//B3LYP/6-31G(d) and SMD(H_2_O)-ωB97XD/def2-TZVP//B3LYP/6-31G(d) (quoted in parentheses) respectively. Bond distances in Å.

As illustrated in Figure 4, isomandapamate **2** can also be obtained through a competing concerted [4 + 2] cycloaddition and stepwise 4 + 2 type pathway. Thus, following the acid-catalyzed conversions of the compounds **11a** and **12a** (Scheme 1a) the stable furanoxonium ion intermediate **Int-13a,** containing an *E*-configured (Δ^6,7^)-double bond, is produced. This intermediate can then either proceed via **TS1a** (Δ*G*^⧧^ of −4.7 kcal mol^–1^) and **TS2a** (Δ*G*^⧧^ of 13.4 kcal mol^–1^) or via **TS3a** (Δ*G*^⧧^ of −16.9 kcal mol^–1^) and **TS4a** (Δ*G*^⧧^ of 1.7 kcal mol^–1^) leading to the tricyclic ring system found in isomandapamate **2** (Figure 4). However, the overall energy barrier for the concerted [4 + 2] cycloaddition route (**Int-13a** → **TS2a**, 35.2 kcal mol^–1^) remains higher than the stepwise 4 + 2 type route (**Int-13a** → **TS4a**, 23.5 kcal mol^–1^), which further indicates that the stepwise transannular C–C bond formation sequence to isomandapamate **2,** similar to that found in mandapamate **1**, is more feasible at room temperature.

### Relative Stereochemistries of the Mandapamates 1 and 2

Our calculations reveal that in the proposed biosynthesis of the mandapamates, the orientations of the oxygen atoms in the furan ring of the furanoxonium ion intermediate **Int-13** will be maintained once the latter is formed. Accordingly, the *Z*-configured (Δ^6,7^) intermediate **Int-13b** points toward (3*R*, 6*S*)-mandapamate **1** and, likewise, the *E*-configured (Δ^6,7^) intermediate **Int-13a** points toward (3*S*, 6*R*)-isomandapamate **2**. Moreover, according to previous conformational analyses, the configuration of the furanoxonium ion intermediate **Int-13** is controlled by the conformational changes shown in the dihydroxy and epoxy compounds **11** and **12** respectively. We can conclude therefore that the initial conformational changes in the macrocyclic furanocembrane precursors dictate the stereoselectivity in the formation of mandapamate **1** and isomandapamate **2**.

### Concerted [4 + 2] Cycloaddition vs Stepwise 4 + 2 Type Pathway

**Figure 5:**
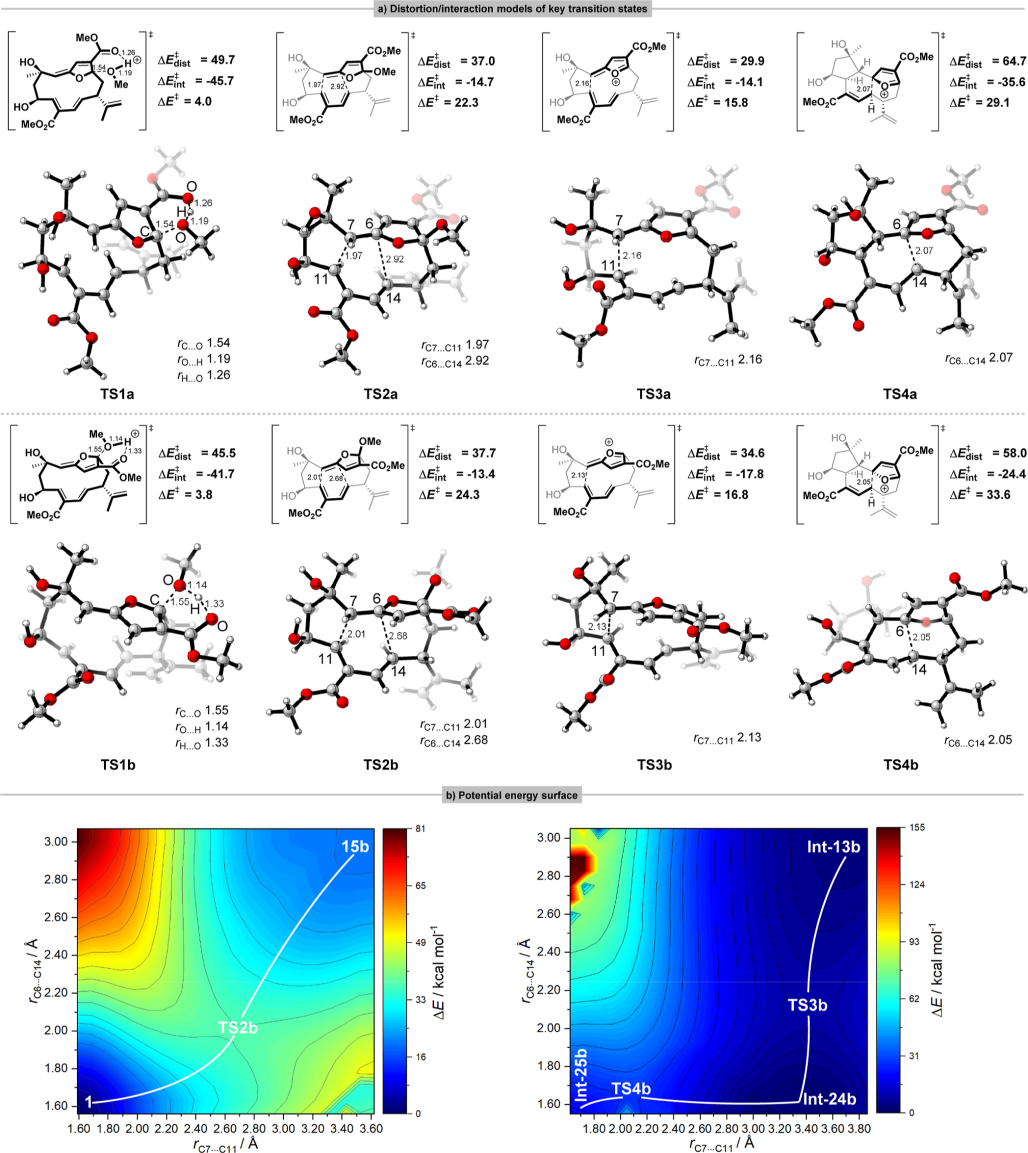
(a) The distortion/interaction analysis of optimized transition states toward mandapamate **1** and isomandapamate **2** at the SMD(H_2_O)-B3LYP-D3(BJ)/def2-TZVP//B3LYP/6-31G(d) level. Energies in kcal mol^–1^. Bond distances in Å. Color scheme of key transition states: H, white; C, gray; O, red. (b) Contour plot of the PES of [4 + 2] concerted and stepwise pathways. Relative energies with respect to the lowest structure energy.

The distortion/interaction model originally conceived for bimolecular reactive systems, can also be applied to intramolecular reactions with a fragmentation scheme.^38^ As displayed in Figure 5a, we computed the strain-related energies of distorting various fragments in the intramolecular transition structures **TS2**, **TS3** and **TS4**. The fragments (bold moieties) are obtained after being separated and filled valences with hydrogen atoms where the covalent bonds have been broken. The distortion/interaction analysis revealed that the distortion energies of **TS3** (**TS3a**: Δ*E*^⧧^_dist_ = 29.9 kcal mol^–1^, **TS3b**: Δ*E*^⧧^_dist_ = 34.6 kcal mol^–1^) representing the first C7–C11 bond formation in the stepwise pathway are lower than that of **TS2** representing concerted pathway (i.e., the concerted formation of the bonds C6–C14 and C7–C11). Compared with **TS2**, the bond distance of the second C6–C14 bond formation in **TS4** are shorter by 0.85 and 0.63 Å respectively, indicating the stronger interaction in the transition states **TS4** (**TS4a**: Δ*E*^⧧^_int_ = −35.6 kcal mol^–1^, **TS4b**: Δ*E*^⧧^_int_ = −24.4 kcal mol^–1^) of the stepwise pathway. Through constrained coordinate scans, we obtained the PES describing the mechanism of formation of the transannular C–C bonds C6–C14 and C7–C11 at the B3LYP/6-31G(d) level. The most striking feature of the [4 + 2] cycloaddition pathway toward mandapamate **1** is that there is only one single saddle point connecting the intermediate **15b** and **1** (Figure 3), and clearly this corresponds to the concerted TS **TS2b** shown in Figure 5b (left). By contrast, the stepwise 4 + 2 type pathway to mandapamate **1** presents two saddle points connecting three species with much lower energy barriers in Figure 5b (right). Thus, the first transannular C–C bond formation occurs between C7 and C11 via TS **TS3b** (Figure 5b, right), and then the second transannular C–C bond forms immediately via TS **TS4b**, leading to formation of the key intermediate **Int-25b** (Figure 3) containing the tricyclic ring system in the natural product. Likewise, the above is also applicable to the pathway toward isomandapamate **2**, due to the presence of the same carbocyclic skeleton, albeit with different orientations of the oxygen-bridged moiety.

## Conclusion

**Scheme 2:**
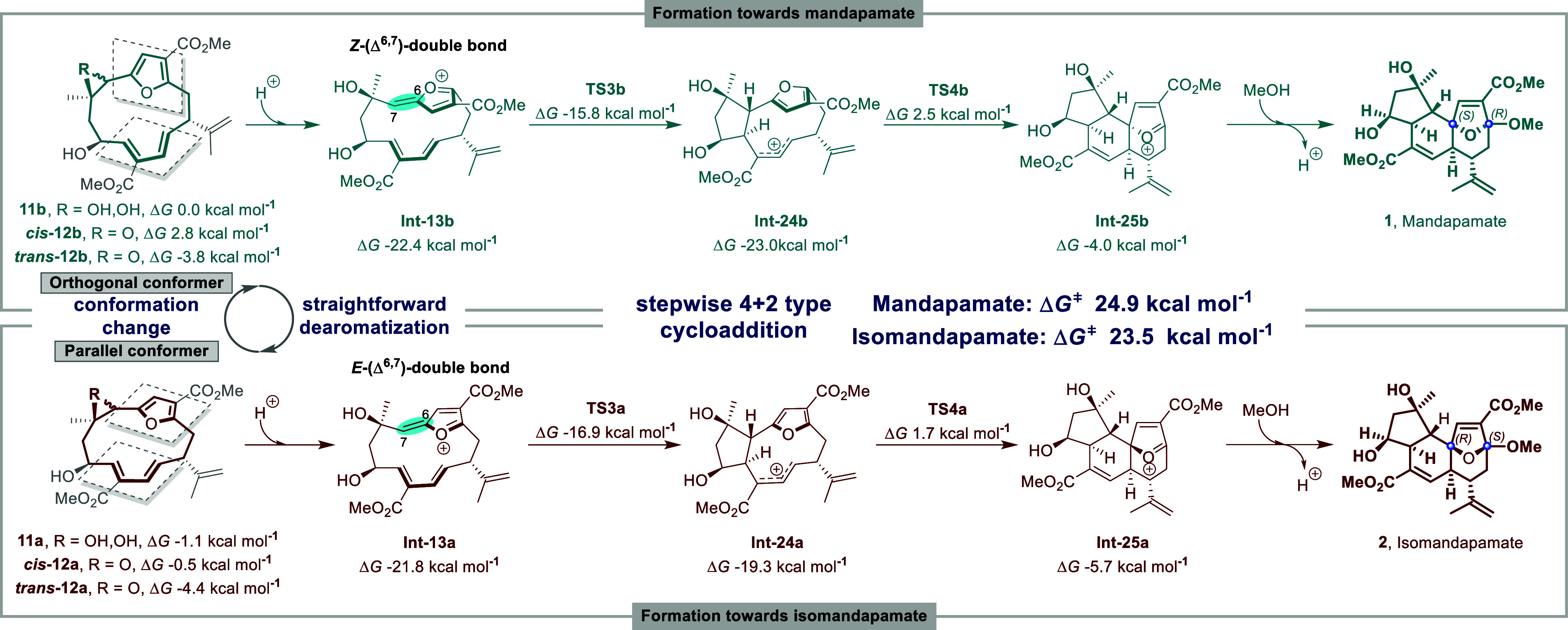
Summary of the Computed Biosyntheses of Mandapamate 1 and Isomandapamate 2

We have explored the concerted and the stepwise pathways leading to the formation of the tricyclic ring systems in the mandapamates **1** and **2** using quantum chemical calculations (Scheme 2). Our calculations reveal that the topological shift between conformers in the macrocyclic precursors to **1** and **2** is significant in controlling which one leads to the (3*R*, 6*S*)-mandapamate **1** and which to the (3*S*, 6*R*)- isomandapamate **2** via *Z*-configured (Δ^6,7^) **Int-13b** and *E*-configured (Δ^6,7^) **Int-13a** respectively. Our study therefore highlights the significance and importance of analyzing the conformations of macrocyclic molecules in order to appreciate their inherent ring-strained reactivity.^39^ The energy barriers between the conformers **Int-13a** and **Int-13b** (ΔΔ*G*_**11a**⇄**11b**_ = 1.1 kcal mol^–1^, ΔΔ*G*_*cis***-12a**⇄*cis***-12b**_ = 3.3 kcal mol^–1^, ΔΔ*G*_*trans***-12a**⇄*trans***-12b**_ = 0.6 kcal mol^–1^) would allow facile conformational changes in conditions in vivo. Comparing the proposed cyclization routes to the ring systems in the mandapamates, calculations demonstrate that a stepwise sequence is energetically favored over an alternative concerted [4 + 2] cycloaddition pathway at room temperature. Our calculations also demonstrate that the stepwise pathway explains the formation of the different stereochemistries found in the naturally occurring isomeric mandapamates **1** and **2**. Our study has provided some new mechanistic insight. However, the actual biosynthetic pathway may differ from that suggested by our calculations. For example, enzymes can alter reaction pathways beyond simply lowering a TS barrier. We can only rely on synthetic strategies in the lab to explore the chemistry of synthesizing macrocyclic precursors as well as biomimetically synthesizing mandapamates **1** and **2**. Synthetic work toward the synthesis of macrocyclic furanocembranoid **11**/**12** for the biomimetic syntheses of mandapamates **1** and **2** is currently being carried out in our laboratory. Progress will be reported in due course.

## Data Availability

The data underlying this study are available in the published article and its Supporting Information

## Supporting Information Available

Details of DFT calculations (PDF)
