# Supplementary material for: Theoretical Study on the Biosynthesis of the Mandapamates: Mechanistic Insights Using Density Functional Theory
Source: J Org Chem. 2024 Sep 9;89(18):12946–56. doi: 10.1021/acs.joc.4c00859 (PMC11421023; doi:10.1021/acs.joc.4c00859)
Supplement: Supplementary file 1 — jo4c00859_si_001.pdf [file jo4c00859_si_001.pdf]

Supporting Information

Theoretical Study on the Biosynthesis of the Mandapamates: Mechanistic Insights Using Density Functional Theory

Di Wang,[a] Gerald Pattenden,[b] Kam Loon Fow,[a] Michael J. Stocks,[c] Jonathan D. Hirst,*[b] and Bencan Tang*[a]

[a] Nottingham Ningbo China Beacons of Excellence Research and Innovation Institute. Key Laboratory for Carbonaceous Waste Processing and Process Intensification Research of Zhejiang Province. Department of Chemical and Environmental Engineering, The University of Nottingham Ningbo China, 199 Taikang East Road, Ningbo 315100 (P. R. China)

[b] School of Chemistry, University of Nottingham, University Park, Nottingham NG7 2RD (UK)

[c] Nottingham University Biodiscovery Institute, School of Pharmacy, University of Nottingham, University Park, Nottingham NG7 2RD (UK)

Corresponding E-mail: bencan.tang@nottingham.edu.cn

jonathan.hirst@nottingham.ac.uk

Table of Contents

1. Computational Methods S1

2. Benchmark Study S1

3. Conformational Analysis of 11 and 12 .... S4

4. Summary of the Calculated Free Energies S7

5. One Possible Side Reaction S11

6. References S12

7. Cartesian Coordinates S14

1. Computational Methods

All DFT calculations were carried out using Gaussian 16 software package[1]. All geometry optimizations were performed with the B3LYP functional[2] using the 6-31G(d)[3] basis set for all atoms. The vibrational frequencies were computed at the same level of theory as for the geometry optimizations to confirm whether each optimized structure is an energy minimum (possessing zero imaginary frequencies) or a transition state (TS, possessing one single imaginary frequency), and to obtain the zero-point vibrational energy (ZPVE) and thermal corrections under 298.15 K and 1 atm pressure. All transition states were confirmed to connect reactants and products by intrinsic reaction coordinate (IRC) calculations. The single point energies for all optimized structures and solvent effects were computed with the B3LYP functional including Grimme's D3 (BJ-damping)[4] dispersion corrections and the ωB97XD[5] functional using the triple-ζ valence def2-TZVP[6] basis set for all atoms. The solvation energies were evaluated by a self-consistent reaction field (SCRF) using the SMD implicit solvent model of water.[7] The 3D graphics of molecules were generated using CYLView.[8] Potential energy surfaces (PES) were produced by OriginPro Learning Edition.

2. Benchmark Study

The calculation started from optimization of the single-crystal X-ray diffraction structures of providencin[9], lophotoxin[10], leptolide[10], pukalide aldehyde[10] and rameswaralide[11]. As a benchmark study, these X-ray structures were optimized at B3LYP/6-31G(d), MPWB1K[12]/6-31G(d), M06-2X[13]/6-31G(d) and ωB97XD/6-31G(d) separately. The root-mean-square deviation (RMSD) between the X-ray structure and the optimized structure of these natural products is displayed in Tables S1 and S2. Comparing different natural products under the same level, leptolide and pukalide aldehyde gave two of the smallest RMSDs at the B3LYP/6-31G(d) level (0.188 Å and 0.195 Å, respectively for all atoms; 0.0900 Å and 0.115 Å, respectively for

non-hydrogen atoms) and at the ωB97XD/6-31G(d) level (0.215 Å and 0.198 Å, respectively for all atoms). Comparing different levels of theory with the same natural product, leptolide gave two of the best results at the B3LYP/6-31G(d) level (0.188 Å for all atoms; 0.0900 Å for non-hydrogen atoms) and at the ωB97XD/6-31G(d) level (0.215 Å for all atoms; 0.112 Å for non-hydrogen atoms). Similarly, pukalide aldehyde also gave two of the best results at the B3LYP/6-31G(d) level (0.195 Å for all atoms; 0.115 Å for non-hydrogen atoms) and at the ωB97XD/6-31G(d) level (0.198 Å for all atoms; 0.138 Å for non-hydrogen atoms). In our assessment of the appropriate level of theory, we bear in mind that X-ray crystallography cannot refine hydrogen atoms in most cases, so hydrogen atoms sometimes are absent or added theoretically on the calculated positions. Furthermore, the skeletons of leptolide and pukalide aldehyde have the closest similarity with our proposed furanocembranoid precursors (Figure S1). Based on the above factors, we decided to use the B3LYP/6-31G(d) level for the geometry optimizations and the single point energies were calculated at the SMD(H_2O)-B3LYP-D3(BJ)/def2-TZVP level and SMD(H_2O)-ωB97XD/def2-TZVP level.

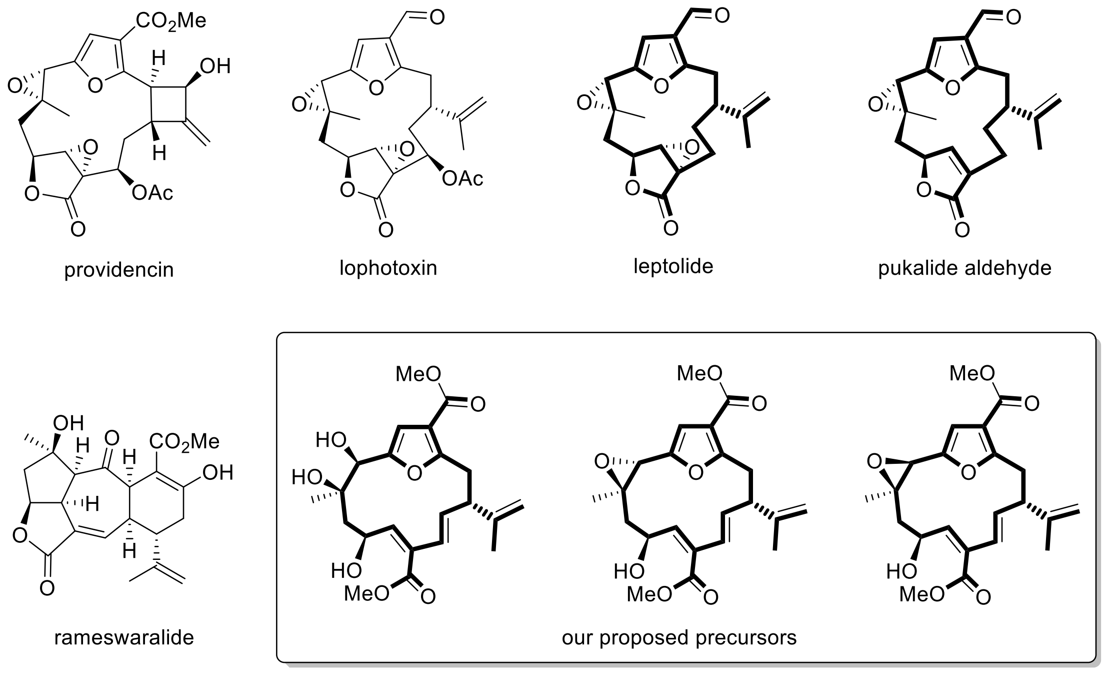

Figure S1. Comparison of different furanocembranoid skeletons.

Table S1. The all-atom root-mean-square deviation (RMSD) in Å between the X-ray structure and the optimized structures of providencin, lophotoxin, leptolide, pukalide aldehyde and rameswaralide.

Natural Products	B3LYP/6-31G(d)	MPWB1K/6-31G(d)	M06-2X/6-31G(d)	ωB97XD/6-31G(d)	Average (μ)	Standard Deviation (σ)
Providencin	0.304	0.242	0.234	0.243	0.256	0.0281
Lophotoxin	0.444	0.389	0.377	0.418	0.407	0.0260
Leptolide	**0.188**	0.229	0.244	**0.215**	0.219	0.0206
Pukalide aldehyde	**0.195**	0.209	0.218	**0.198**	0.205	0.00914
Rameswaralide	0.243	0.292	0.224	0.217	0.244	0.0293

Table S2. The non-hydrogen atom RMSD in Å between the X-ray structure and the optimized structures of providencin, lophotoxin, leptolide, pukalide aldehyde and rameswaralide.

Natural Products	B3LYP/6-31G(d)	MPWB1K/6-31G(d)	M06-2X/6-31G(d)	ωB97XD/6-31G(d)	Average (μ)	Standard Deviation (σ)
Providencin	0.184	0.159	0.152	0.143	0.160	0.0152
Lophotoxin	0.364	0.312	0.299	0.340	0.329	0.0252
Leptolide	**0.0900**	0.138	0.148	**0.112**	0.122	0.0227
Pukalide aldehyde	**0.115**	0.158	0.167	**0.138**	0.145	0.0200
Rameswaralide	0.130	0.196	0.0765	0.0861	0.122	0.0472

In addition, B3LYP, M06-2X and ωB97XD functionals were also applied to optimize the key transition states and the corresponding intermediates involved in the pathways. The single point energies were computed with B3LYP-D3(BJ), M06-2X and ωB97XD functionals separately using the def2-TZVP basis set as well as including a SMD implicit solvent model of water. The computed activation Gibbs free energies of each transition state are listed in the Table S3. Different density functionals provided similar results and conclusions, indicating that the results with B3LYP-D3(BJ) and ωB97XD functionals are suitable and reliable. Furthermore, B3LYP/6-31G(d) level is generally applied to achieve the best balance between accuracy and computational costs for the geometry optimization. However, an abnormal deviation (red

marked) may indicate that the M06-2X functional would be not suitable for this system and thus was ruled out in this work.

Table S3. The computed activation Gibbs free energies (in kcal mol^{-1}) with different density functionals.

Density functional	B3LYP-D3(BJ)[a]	ωB97XD[b]	M06-2X[c]	ωB97XD[d]
$\Delta G^{‡}$(**TS1a**)	10.2	7.4	3.4	6.1
$\Delta G^{‡}$(**TS1b**)	9.2	6.9	0.0	8.0
$\Delta G^{‡}$(**TS2a**)	15.6	19.9	19.0	18.8
$\Delta G^{‡}$(**TS2b**)	16.0	20.5	18.8	18.8
$\Delta G^{‡}$(**TS3a**)	4.9	6.8	4.0	6.7
$\Delta G^{‡}$(**TS3b**)	6.6	9.0	8.7	8.9
$\Delta G^{‡}$(**TS4a**)	21.0	18.8	19.3	21.0
$\Delta G^{‡}$(**TS4b**)	25.5	24.5	21.4	23.4

[a]Computed at the SMD(H_2O)/B3LYP-D3(BJ)/def2-TZVP//B3LYP/6-31G(d) level.
[b]Computed at the SMD(H_2O)/ωB97XD/def2-TZVP//B3LYP/6-31G(d) level.
[c]Computed at the SMD(H_2O)/M06-2X/def2-TZVP//M06-2X/6-31G(d) level.
[d]Computed at the SMD(H_2O)/ωB97XD/def2-TZVP//ωB97XD/6-31G(d) level.

3. Conformational Analysis of 11 and 12

In the initial steps, conformational analyses were carried out *via* random searching in the GMMX 3.1 module of Gaussian 16 using the MMFF94 force field. Initial selection of the related conformations relied on a combination of guidance from energy data, chemical intuition and the use of relaxed coordinate scans. Rotation about the furan ring was considered to be an important conformational feature and a relaxed coordinate scan was performed at the DFT B3LYP/6-31G(d) level of theory incrementing the O−C6−C7−C8 dihedral angles for each of the three model furanocembranoids (**11**, ***cis*-12** and ***trans*-12**). The O−C6−C7−C8 dihedral angle was considered, for **11** in Figure S2, the scanning starts from $\Phi_{O-C6-C7-C8}$ = -126.6° with 10° increments. For ***cis*-12** in Figure S3, the scanning starts from $\Phi_{O-C6-C7-C8}$ = 83.6° with -10° increments. For ***trans*-12** in Figure S4, the scanning starts from $\Phi_{O-C6-C7-C8}$ = -67.5° with -10°

increments. With the scanning curves in hand, the initial guess of the TS geometry was taken from the local maxima (energy inflexion) on the curves and the Berny geometry optimization algorithm was used for the optimizations to the transition states. Although each TS structure possessed only one imaginary vibrational frequency, using intrinsic reaction coordinate (IRC) calculations to connect the surrounding minima was unsuccessful. We speculate that the low imaginary vibrational frequency of each transition state only represents the vibration of the macrocyclic skeleton and result in the failure of the IRC calculations. However, through manual displacement of every imaginary vibrational frequency vector in both the positive (+5%) and negative (-5%) direction, these two structures could be optimized to the local minima to afford the expected parallel and orthogonal conformers. Therefore, this confirms that the obtained transition states were reasonable.

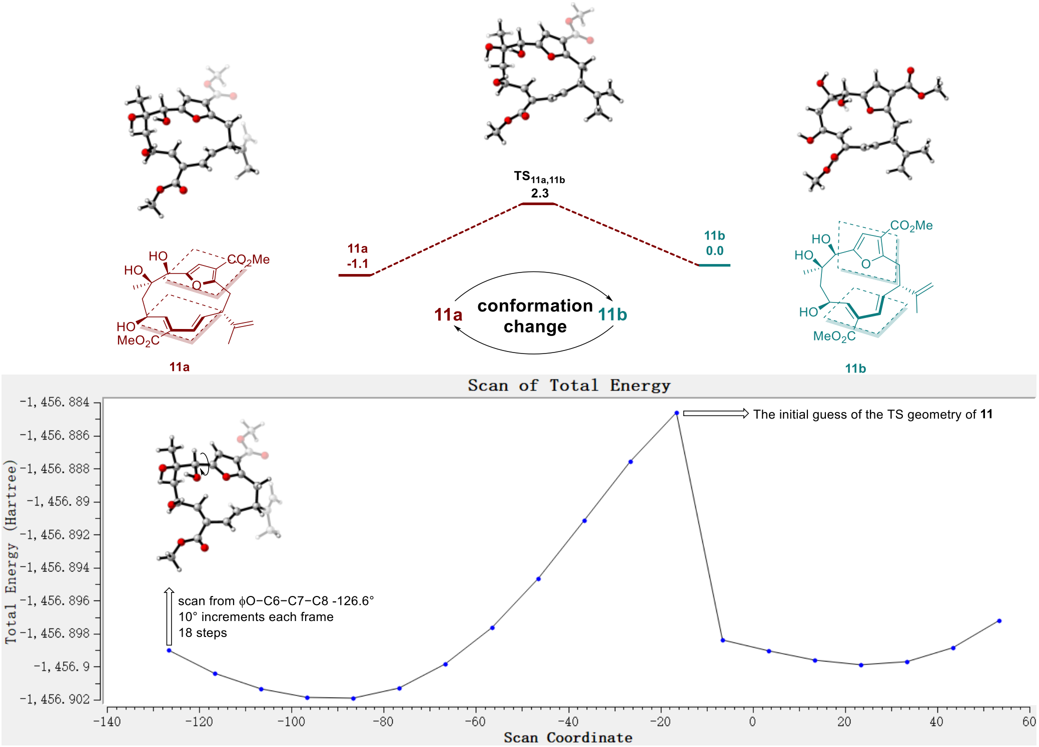

Figure S2. The conformation search of **11**. The relaxed coordinate scan was at B3LYP/6-31G(d) level. Geometry optimized with B3LYP/6-31G(d) in gas phase. Free energy calculated with SMD(H_2O)-B3LYP-D3(BJ)/def2-TZVP//B3LYP/6-31G(d).

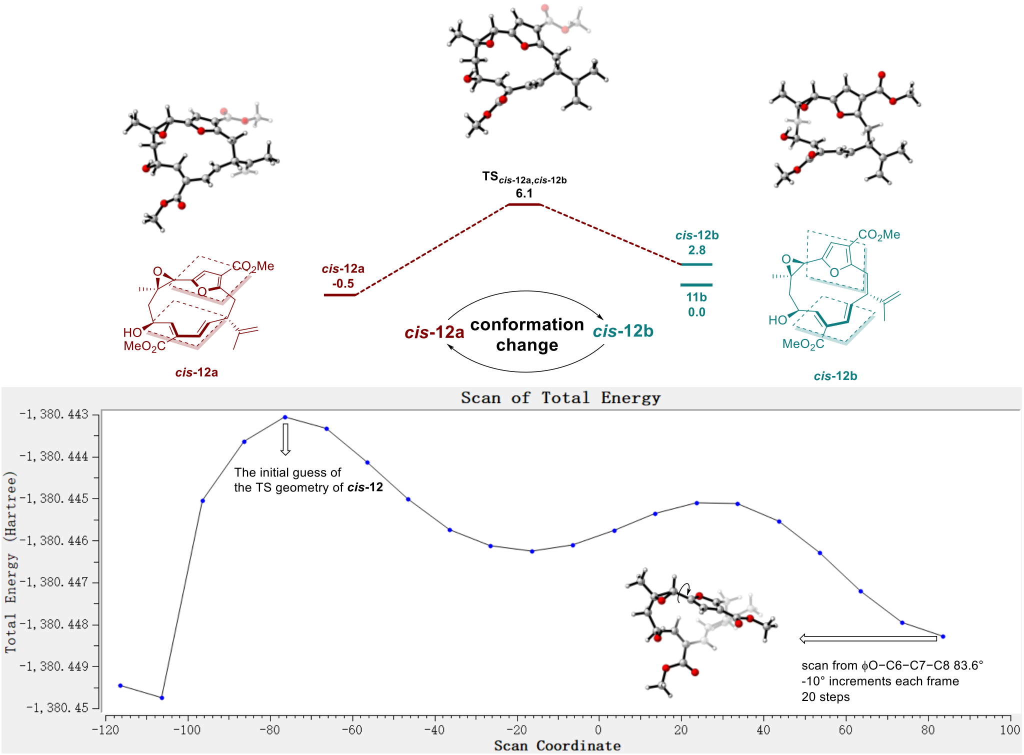

Figure S3. The conformation search of ***cis*-12**. The relaxed coordinate scan was at the B3LYP/6-31G(d) level. Geometry optimized with B3LYP/6-31G(d) in gas phase. Free energy calculated with SMD(H_2O)-B3LYP-D3(BJ)/def2-TZVP//B3LYP/6-31G(d).

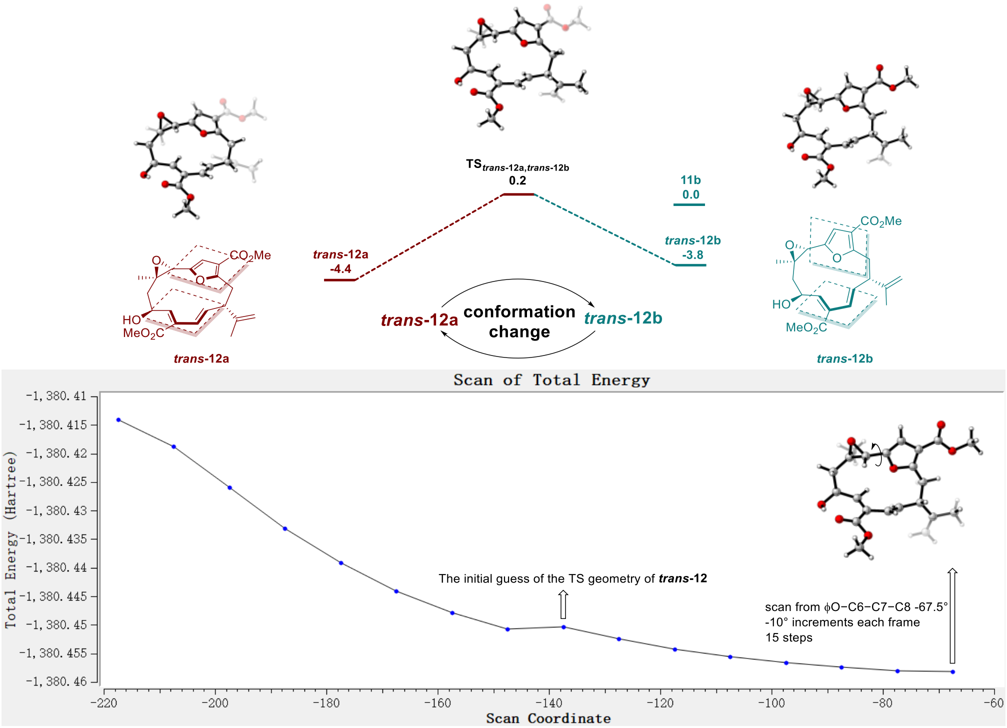

Figure S4. The conformation search of ***trans*-12**. The relaxed coordinate scan was at the B3LYP/6-31G(d) level. Geometry optimized with B3LYP/6-31G(d) in gas phase. Free energy calculated with SMD(H_2O)-B3LYP-D3(BJ)/def2-TZVP//B3LYP/6-31G(d).

4. Summary of the Calculated Free Energies

Table S4. Calculated absolute energies and free energies (in Hartrees) of all structures at the SMD(H_2O)-B3LYP-D3(BJ)/def2-TZVP//B3LYP/6-31G(d) and SMD(H_2O)-ωB97XD/def2-TZVP//B3LYP/6-31G(d).

Structure	E_{elec} [a]	ZPE [b]	H_{corr} [c]	G_{corr} [d]	$E_{sol-B3LYP-D3(BJ)}$ [e]	$E_{sol-ωB97XD}$ [f]	IF [g] (cm^{-1})
**11a**	-1456.899004	0.479618	-1456.388003	-1456.481472	-1457.634647	-1457.054588	
**TS$_{11a,11b}$**	-1456.893373	0.478714	-1456.383866	-1456.47437	-1457.630840	-1457.051560	-17.86
**11b**	-1456.875196	0.476472	-1456.366053	-1456.463084	-1457.627545	-1457.049064	
***cis*-12a**	-1380.449755	0.450519	-1379.969086	-1380.06025	-1381.132851	-1380.580220	
**TS$_{cis\text{-}12a,\ cis\text{-}12b}$**	-1380.443047	0.449663	-1379.963878	-1380.05273	-1381.123100	-1380.570238	-21.92
***cis*-12b**	-1380.446261	0.450455	-1379.965618	-1380.057537	-1381.126778	-1380.573052	
***trans*-12a**	-1380.455091	0.450418	-1379.974556	-1380.065934	-1381.138752	-1380.584993	
**TS$_{trans\text{-}12a,\ trans\text{-}12b}$**	-1380.452166	0.450441	-1379.972472	-1380.061034	-1381.133415	-1380.579534	-41.63
***trans*-12b**	-1380.458198	0.450586	-1379.977604	-1380.068767	-1381.138063	-1380.584836	
**Int-13a**	-1380.839796	0.462358	-1380.347317	-1380.437417	-1381.582400	-1381.017292	
**Int-13b**	-1380.825318	0.460674	-1380.333675	-1380.425788	-1381.580537	-1381.017644	
**Int-14a**	-1496.567400	0.515669	-1496.016704	-1496.119777	-1497.373327	-1496.764057	
**Int-14b**	-1496.558019	0.514655	-1496.007845	-1496.111159	-1497.368565	-1496.760799	
**TS1a**	-1496.548435	0.514262	-1496.00156	-1496.096558	-1497.361279	-1496.756551	-938.40
**TS1b**	-1496.541358	0.513459	-1495.994834	-1496.089746	-1497.358612	-1496.754509	-677.12
**Int-23a**	-1496.555359	0.518564	-1496.003871	-1496.099517	-1497.366926	-1496.763214	
**Int-23b**	-1496.551513	0.517646	-1496.000464	-1496.096129	-1497.365416	-1496.762136	
**15a**	-1496.190788	0.506088	-1495.651832	-1495.747567	-1496.945854	-1496.344047	
**15b**	-1496.182761	0.504872	-1495.644406	-1495.740964	-1496.944224	-1496.343619	
**TS2a**	-1496.167703	0.505993	-1495.630085	-1495.722304	-1496.923241	-1496.314588	-362.62
**TS2b**	-1496.155307	0.504357	-1495.618372	-1495.713596	-1496.918701	-1496.310908	-401.99
**TS3a**	-1380.825280	0.461454	-1380.334055	-1380.422892	-1381.574743	-1381.006435	-260.89
**TS3b**	-1380.817594	0.460458	-1380.326917	-1380.417385	-1381.570801	-1381.003884	-270.67
**Int-24a**	-1380.834233	0.463591	-1380.34087	-1380.429978	-1381.580395	-1381.023172	
**Int-24b**	-1380.828381	0.46165	-1380.336118	-1380.42804	-1381.582347	-1381.026027	

**TS4a**	-1380.791180	0.462423	-1380.299719	-1380.387446	-1381.546384	-1380.992574	-307.53
**TS4b**	-1380.787655	0.461537	-1380.296567	-1380.385801	-1381.543300	-1380.988492	-326.54
**Int-25a**	-1380.800316	0.463599	-1380.307334	-1380.396003	-1381.558744	-1381.011493	
**Int-25b**	-1380.799820	0.462926	-1380.307158	-1380.396519	-1381.555091	-1381.007968	
**Int-26a**	-1496.577014	0.521014	-1496.024124	-1496.117349	-1497.398328	-1496.812869	
**Int-26b**	-1496.578741	0.520423	-1496.026003	-1496.120232	-1497.396952	-1496.811822	
**1**	-1496.209749	0.507614	-1495.669718	-1495.76471	-1496.972740	-1496.388370	
**2**	-1496.213967	0.508718	-1495.673419	-1495.767629	-1496.973771	-1496.389021	
**H_2O**	-76.408954	0.021167	-76.384008	-76.405454	-76.476410	-76.450691	
**H_3O^+**	-76.689085	0.034307	-76.650943	-76.672897	-76.891926	-76.870006	
**CH_3OH**	-115.714406	0.051474	-115.658699	-115.685652	-115.785348	-115.741649	

[a]The electronic energy calculated at the B3LYP/6-31G(d). [b]Zero-point correction energy calculated at the B3LYP/6-31G(d). [c]The thermal correction to enthalpy calculated at the B3LYP/6-31G(d). [d]The thermal correction to Gibbs free energy calculated at the B3LYP/6-31G(d). [e]The electronic energy calculated at the SMD(H_2O)-B3LYP-D3(BJ)/def2-TZVP. [f]The electronic energy calculated at the SMD(H_2O)-ωB97XD/def2-TZVP. [g]Imaginary frequencies for the transition states.

All Gibbs free energies discussed in the manuscript were corrected using the quasi-rigid rotor-harmonic oscillator (QRRHO) approach proposed by Grimme[14] and implemented in the GoodVibes code.[15]

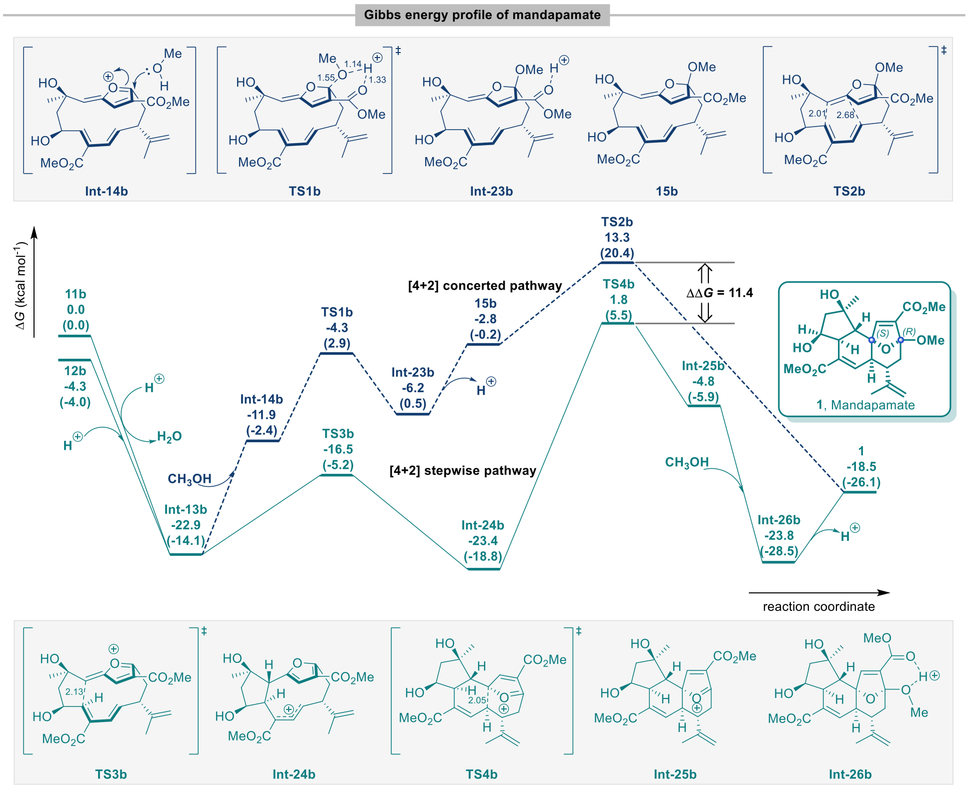

Figure S5. Energy profile for the formation of mandapamate **1**. All Gibbs free energies were calculated at the SMD(H_2O)-B3LYP-D3(BJ)/def2-TZVP//B3LYP/6-31G(d) and SMD(H_2O)-ωB97XD/def2-TZVP//B3LYP/6-31G(d) level (quoted in parentheses) and corrected using GoodVibes code.

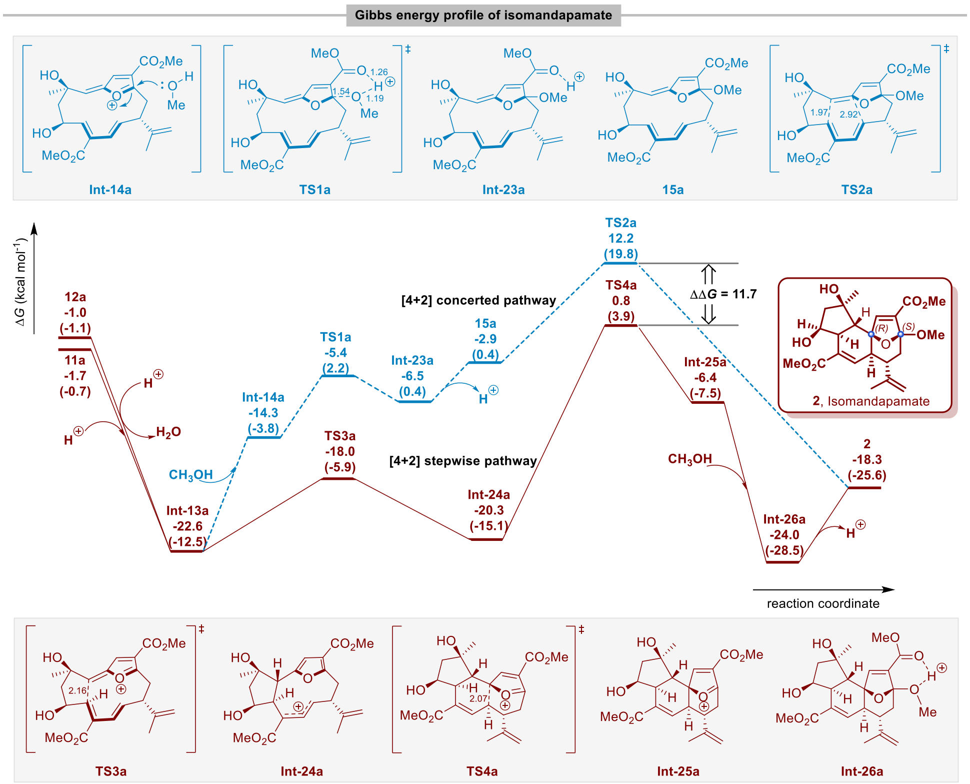

Figure S6. Energy profile for the formation of isomandapamate **2**. All Gibbs free energies were calculated at the SMD(H_2O)-B3LYP-D3(BJ)/def2-TZVP//B3LYP/6-31G(d) and SMD(H_2O)-ωB97XD/def2-TZVP//B3LYP/6-31G(d) level (quoted in parentheses) and corrected using GoodVibes code.

Table S5. Corrected Gibbs free energies (in Hartrees) of all structures using GoodVibes code.

Structure	$G_{sol\text{-}B3LYP\text{-}D3(BJ)}$[a]	$G_{sol\text{-}\omega B97XD}$[b]
**11a**	-1457.223052	-1456.642993
**TS$_{11a,11b}$**	-1457.218833	-1456.639553
**11b**	-1457.220319	-1456.641837
***cis*-12a**	-1380.748486	-1380.195855
**TS$_{cis\text{-}12a,\ cis\text{-}12b}$**	-1380.738716	-1380.185855
***cis*-12b**	-1380.742712	-1380.188986
***trans*-12a**	-1380.754548	-1380.200789
**TS$_{trans\text{-}12a,\ trans\text{-}12b}$**	-1380.748198	-1380.194316
***trans*-12b**	-1380.753716	-1380.200489
**Int-13a**	-1381.186082	-1380.620974
**Int-13b**	-1381.186511	-1380.623618
**Int-14a**	-1496.930563	-1496.321293
**Int-14b**	-1496.926852	-1496.319087
**TS1a**	-1496.916431	-1496.311703
**TS1b**	-1496.914632	-1496.310529
**Int-23a**	-1496.918185	-1496.314473
**Int-23b**	-1496.917681	-1496.314401
**15a**	-1496.509380	-1495.907572
**15b**	-1496.509179	-1495.908574
**TS2a**	-1496.485306	-1495.876653
**TS2b**	-1496.483473	-1495.875680
**TS3a**	-1381.178775	-1380.610467
**TS3b**	-1381.176294	-1380.609376
**Int-24a**	-1381.182339	-1380.625117
**Int-24b**	-1381.187303	-1380.630983
**TS4a**	-1381.148740	-1380.594930
**TS4b**	-1381.147159	-1380.592352
**Int-25a**	-1381.160275	-1380.613024

**Int-25b**	-1381.157642	-1380.610518
**Int-26a**	-1496.946048	-1496.360588
**Int-26b**	-1496.945763	-1496.360632
**1**	-1496.534249	-1495.949879
**2**	-1496.533811	-1495.949061
**H_2O**	-76.473396	-76.447677
**H_3O^+**	-76.876529	-76.854609
**CH_3OH**	-115.757790	-115.714092

[a]Calculated and corrected at the SMD(H_2O)-B3LYP-D3(BJ)/def2-TZVP//B3LYP/6-31G(d) level. [b]Calculated and corrected at the SMD(H_2O)-ωB97XD/def2-TZVP//B3LYP/6-31G(d).

5. One Possible Side Reaction

The proposed reaction mechanism was based on a hypothetical precursor. So, multiple side reactions are possible from the proposed precursor that would lead to various side products. For example, transannular cycloaddition with the furan ring and the *exo* C(15)=C is a possibility. However, our calculations revealed that the assoicated transition state has a very high activation energy barrier of over 59.0 kcal mol^{-1}, which rules out this pathway (Figure S7).

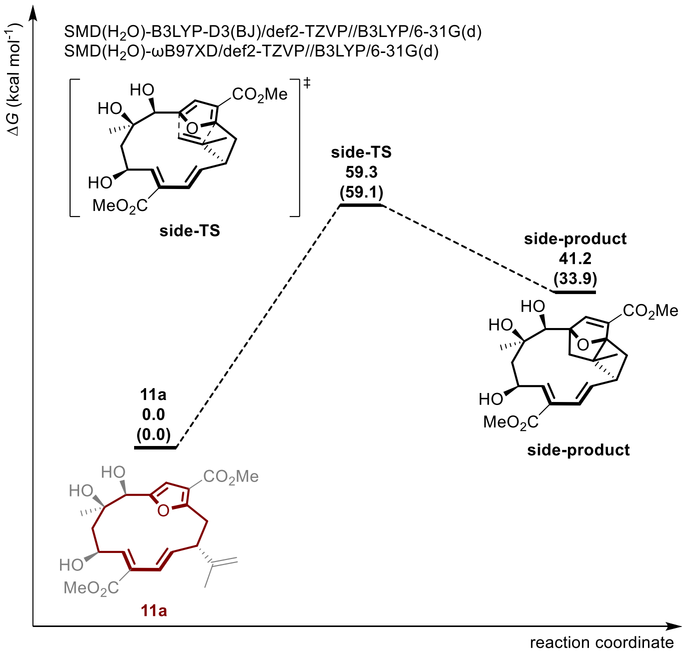

Figure S7. Energy profile for the formation of **side-product** *via* **side-TS**.

6. References

[1] Gaussian 16, Revision A.03, M. J. Frisch, G. W. Trucks, H. B. Schlegel, G. E. Scuseria, M. A. Robb, J. R. Cheeseman, G. Scalmani, V. Barone, G. A. Petersson, H. Nakatsuji, X. Li, M. Caricato, A. V. Marenich, J. Bloino, B. G. Janesko, R. Gomperts, B. Mennucci, H. P. Hratchian, J. V. Ortiz, A. F. Izmaylov, J. L. Sonnenberg, D. Williams-Young, F. Ding, F. Lipparini, F. Egidi, J. Goings, B. Peng, A. Petrone, T. Henderson, D. Ranasinghe, V. G. Zakrzewski, J. Gao, N. Rega, G. Zheng, W. Liang, M. Hada, M. Ehara, K. Toyota, R. Fukuda, J. Hasegawa, M. Ishida, T. Nakajima, Y. Honda, O. Kitao, H. Nakai, T. Vreven, K. Throssell, J. A. Montgomery, Jr., J. E. Peralta, F. Ogliaro, M. J. Bearpark, J. J. Heyd, E. N. Brothers, K. N. Kudin, V. N. Staroverov, T. A. Keith, R. Kobayashi, J. Normand, K. Raghavachari, A. P. Rendell, J. C. Burant, S. S. Iyengar, J. Tomasi, M. Cossi, J. M. Millam, M. Klene, C. Adamo, R. Cammi, J. W. Ochterski, R. L. Martin, K. Morokuma, O. Farkas, J. B. Foresman, and D. J. Fox, Gaussian, Inc., Wallingford CT, 2016.

[2] (a) A. D. Becke, *J. Chem. Phys.*, **1993**, *98*, 5648. (b) C. Lee, W. Yang, R. G. Parr, *Phys. Rev. B*, **1988**, *37*, 785.

[3] (a) R. Ditchfield, W. J. Hehre, J. A. Pople, *J. Chem. Phys.*, **1971**, *54*, 724. (b) W. J. Hehre, R. Ditchfield, J. A. Pople, *J. Chem. Phys.*, **1972**, *56*, 2257. (c) R. Krishnan, J. S. Binkley, R. Seeger, J. A. Pople, *J. Chem. Phys.*, **1980**, *72*, 650. (d) P. C. Hariharan, J. A. Pople, *Theoret. Chim. Acta*, **1973**, *28*, 213.

[4] (a) S. Grimme, J. Antony, S. Ehrlich, H. Krieg, *J. Chem. Phys.*, **2010**, *132*, 154104. (b) S. Grimme, S. Ehrlich, L. Goerigk, *J. Comput. Chem.*, **2011**, *32*, 1456.

[5] J.-D. Chai, M. Head-Gordon, *Phys. Chem. Chem. Phys.*, **2008**, *10*, 6615.

[6] F. Weigend, R. Ahlrichs, *Phys. Chem. Chem. Phys.*, **2005**, *7*, 3297.

[7] (a) J. Tomasi, B. Mennucci, R. Cammi, *Chem. Rev.*, **2005**, *105*, 2999. (b) A. V. Marenich, C. J. Cramer, D. G. Truhlar, *J. Phys. Chem. B.*, **2009**, *113*, 6378.

[8] CYLview20; C. Y. Legault, Université de Sherbrooke, **2020** (http://www.cylview.org)

[9] J. Marrero, A. D. Rodríguez, P. Baran, R. G. Raptis, *Org. Lett.*, **2003**, *5*, 2551.

[10] M. Gutiérrez, T. L. Capson, H. M. Guzmán, J. González, E. Ortega-Barría, E. Quiñoá, R.

Riguera, *J. Nat. Prod.*, **2005**, *68*, 614.

[11] B. R. Chitturi, V. B. Tatipamula, C. B. Dokuburra, U. K. Mangamuri, V. R. Tuniki, S. V. Kalivendi, R. A. Bunce, V. Yenamandra, *Tetrahedron*, **2016**, *72*, 1933.

[12] Y. Zhao, D. G. Truhlar, *J. Phys. Chem. A*, **2004**, *108*, 6908.

[13] Y. Zhao, D. G. Truhlar, *Theor. Chem. Acc.*, **2008**, *120*, 215.

[14] S. Grimme, *Chem. -Eur. J.*, **2012**, *18*, 9955.

[15] G. Luchini, J. V. Alegre-Requena, I. Funes-Ardoiz, R. S. Paton, GoodVibes: Automated Thermochemistry for Heterogeneous Computational Chemistry Data. *F1000Research*, **2020**, *9*, 291. DOI: 10.12688/f1000research.22758.1

7. Cartesian Coordinates

11a

C	-1.75555200	0.24742800	0.70110300
C	-2.02294300	1.49600400	0.26375400
C	-0.93417700	2.31550200	-0.30343800
C	0.34951000	2.19761500	0.05752400
C	0.57085400	-1.70854600	-0.75432300
C	-0.67780800	-2.50110300	-1.06113400
C	1.54211700	2.67451300	-0.73242500
C	2.20812000	1.44271900	-1.48141900
O	0.67435700	-0.43896900	-1.27423700
C	1.90827600	0.06248100	-0.97814000
C	2.61177500	-0.89544400	-0.27889100
C	1.73912000	-2.03071800	-0.13604400
C	-1.50209600	-3.12140200	0.13748500
C	-1.90510600	-2.19254000	1.31511000
C	2.46716200	3.51366200	0.14718900
H	0.60478900	1.60079200	0.93365000
H	-0.71041000	-0.03350300	0.70049200
C	-0.75057100	-4.33138500	0.70251700
O	-2.69138100	-3.64605000	-0.48117600
H	-0.35852500	-3.38157700	-1.63969700
O	-3.73662500	2.78632700	-0.80324500
C	1.90653000	4.87711100	0.48352900
C	3.64877000	3.10883800	0.62148700
C	4.01266100	-0.81680000	0.15216000
O	4.79058500	0.08944500	-0.09380900
O	4.36984900	-1.91337600	0.86351100
C	5.73557600	-1.93845800	1.30613300
H	-1.22271000	2.95181900	-1.13827200
H	1.17110400	3.33768200	-1.52571600
H	1.84802200	1.47604400	-2.51577000
H	3.29232600	1.56261600	-1.50627800
H	1.99858400	-2.96540800	0.33645600
H	-2.52312300	-2.82196600	1.96623600
H	-1.01214900	-1.93064100	1.89527900
H	-1.36823100	-4.82781600	1.45672600
H	-0.54556200	-5.05665900	-0.09210500
H	0.19602700	-4.04080800	1.16857000
H	-3.37871400	-2.95897000	-0.35690000
H	1.76354600	5.47993800	-0.42454200
H	0.92163800	4.79565200	0.96103600
H	2.57025400	5.42893100	1.15567200
H	4.23417700	3.76412700	1.26294600
H	4.08402700	2.14019800	0.40096200
H	5.84681600	-2.87502400	1.85343200
H	6.41788200	-1.90840100	0.45244100
H	5.94488400	-1.08538200	1.95696900
C	-2.69557200	-0.89377300	1.00563100
H	-3.32888400	-0.65879800	1.86588700
C	-3.40819200	2.03643800	0.09454300
O	-3.61905200	-1.08551900	-0.09081600
H	-3.06386500	-0.97073600	-0.89053200
O	-4.24731400	1.62291100	1.06570300
C	-5.61569600	2.03100800	0.90858900
H	-5.68912400	3.12023400	0.85786200
H	-6.03695900	1.59903900	-0.00305100
H	-6.13626500	1.64964500	1.78743500
O	-1.53218400	-1.72134100	-1.90238800
H	-2.18199000	-2.37736800	-2.21316100

$TS_{11a,11b}$

C	1.77823900	-0.03654400	-0.88602900
C	2.24033100	1.15038600	-0.44068600
C	1.27163300	2.10719900	0.12368000
C	0.03393000	2.30627500	-0.34195800
C	-0.90862600	-1.54142500	0.79357100
C	0.29055400	-2.35805000	1.20872600
C	-1.11732400	2.80516500	0.50466300
C	-2.30653000	1.82186800	0.37817700
O	-0.85100200	-0.16320600	0.85900600
C	-2.05220400	0.33997200	0.46294700
C	-2.89323600	-0.70880100	0.15797800
C	-2.15008100	-1.91609800	0.38335000
C	1.02898600	-3.23126200	0.11683800
C	1.54383200	-2.53283000	-1.17285100
C	-1.51187300	4.25048100	0.18500400
H	-0.23189500	1.94930100	-1.33847900
H	0.70159500	-0.12632600	-0.95573400
C	0.12420400	-4.39790800	-0.29567300
O	2.13778200	-3.81761500	0.82178000
H	-0.08685000	-3.11114700	1.91604800
O	4.10502400	2.01817000	0.79134200
C	-0.44114400	5.27027100	0.49428400
C	-2.69667000	4.62154400	-0.31164800
C	-4.27426500	-0.57269400	-0.31320600
O	-4.86094300	0.48159100	-0.48888800
O	-4.84335800	-1.78251500	-0.54268500
C	-6.20066400	-1.74015000	-1.00683000
H	1.56357500	2.50880800	1.09378400
H	-0.77190200	2.79702700	1.54854800
H	-3.07011700	2.08837100	1.12018200
H	-2.80108400	1.97405600	-0.58683300
H	-2.52361300	-2.92152900	0.26222300
H	2.04982300	-3.32691100	-1.73449800
H	0.68786100	-2.22081600	-1.78336000
H	0.67347900	-5.06934100	-0.96214100
H	-0.17625800	-4.97588600	0.58501100
H	-0.77407900	-4.05049500	-0.81485700
H	2.91612500	-3.26515800	0.60459200
H	-0.18622900	5.26114500	1.56336400
H	0.48540200	5.05118100	-0.04976600
H	-0.76496500	6.28186700	0.23258900
H	-2.90873100	5.66896800	-0.51158400
H	-3.49990300	3.92667200	-0.53296900
H	-6.49885400	-2.78165400	-1.13204100
H	-6.84315700	-1.24188700	-0.27579000
H	-6.26687800	-1.20485300	-1.95797900
C	2.52707100	-1.33809600	-1.04309700
H	3.17135000	-1.32161500	-1.92828600
C	3.68888600	1.42429800	-0.18320900
O	3.43214000	-1.52252900	0.06963200
H	2.91373500	-1.21773800	0.84312800
O	4.47670300	0.96614600	-1.17686000
C	5.88889200	1.09879700	-0.94905700
H	6.15214100	2.14150000	-0.75518400
H	6.19056700	0.48516600	-0.09586800
H	6.36662200	0.74327400	-1.86246100
O	1.22416200	-1.53021600	1.90952100
H	1.78452500	-2.17496400	2.37573500

11b

C	2.20723300	0.06577200	-0.60082500
C	2.25292300	1.16584200	0.17197700
C	0.97207700	1.84007900	0.48028000
C	0.00670800	2.01385600	-0.43120300
C	-0.83893400	-1.99105100	0.24018500

C	0.45407700	-2.69202400	0.55801400
C	-1.44369000	2.30706600	-0.15699600
C	-2.30585200	1.16152400	-0.80484600
O	-0.83060600	-0.71776900	-0.28663600
C	-2.09614400	-0.21461000	-0.25616100
C	-2.93571800	-1.17151700	0.27701700
C	-2.11446700	-2.30794300	0.59156800
C	-1.92737100	3.66967900	-0.65874600
C	-3.36208900	4.03481500	-0.35150800
C	-1.11996500	4.51930500	-1.30131100
H	0.24342000	1.79970500	-1.47441200
C	1.49567300	-3.03574100	-0.59438100
C	2.88710200	-2.36972500	-0.40403800
C	3.22010600	-0.96420600	-0.97787400
H	1.22159700	-0.18817300	-0.96759000
H	0.13065400	-3.67890000	0.92335700
O	1.82493500	-4.43472100	-0.43927300
C	0.90915400	-2.84868200	-1.99996100
O	3.52437700	1.68848900	2.13277100
H	3.21107400	-1.03344800	-2.08188800
C	-4.38503900	-1.15915900	0.53172000
O	-4.99224000	-2.10211600	1.00178100
O	-4.98615500	0.00947800	0.19424600
C	-6.40266200	0.05619300	0.43234600
H	0.80750700	2.09626500	1.52728600
H	-1.61963300	2.27482000	0.92882000
H	-3.36721800	1.38202000	-0.69649000
H	-2.08794200	1.15618000	-1.88268700
H	-2.46405500	-3.22377600	1.04649200
H	-4.07880600	3.42962600	-0.92168300
H	-3.59221600	3.87594100	0.71091000
H	-3.55877900	5.08499300	-0.58696600
H	-1.47514700	5.49198500	-1.63274000
H	-0.07920700	4.28622100	-1.50267400
H	3.58739400	-3.07181000	-0.87577200
H	3.11313700	-2.34251000	0.66261400
H	1.02568700	-4.94823300	-0.64124900
H	0.66067500	-1.80991900	-2.22950700
H	1.62744900	-3.21155500	-2.74186200
H	-0.01458900	-3.43230500	-2.10775200
H	-6.71881400	1.04929600	0.11171800
H	-6.91528800	-0.71660100	-0.14626100
H	-6.61909100	-0.09289700	1.49332500
O	4.51857000	-0.58834300	-0.53508400
H	5.08977900	-1.36835400	-0.61150500
C	3.45151100	1.66882600	0.92058100
O	4.38609200	2.18750200	0.10292800
C	5.57592800	2.65185000	0.75609600
H	6.20308500	3.06203700	-0.03632700
H	5.33838500	3.42009200	1.49705800
H	6.08374300	1.82090400	1.25349100
O	1.14694200	-2.00477400	1.59769400
H	0.50851000	-1.81448200	2.30326800

cis-12a

C	1.84525000	0.28485600	-0.52275700
C	1.85137400	1.55959200	-0.08202100
C	0.62210800	2.12541600	0.51528000
C	-0.60659400	1.76461300	0.12363800
C	-0.23908300	-2.18375000	0.57732000
C	1.02584200	-2.88427900	0.91572600
C	-1.88917900	1.97262700	0.87749300
C	-2.23977300	0.62002600	1.64668400
O	-0.56290400	-1.09024500	1.34972900
C	-1.79413900	-0.64066700	0.97436400
C	-2.27847700	-1.46116300	-0.02194400
C	-1.26333700	-2.44794100	-0.27665400
C	-3.04599100	2.49841600	0.03160800
C	-4.32242900	2.81580100	0.77587300
C	-2.93616100	2.74529100	-1.27811500
H	-0.69808600	1.12684000	-0.75436200
C	2.28542300	-2.85549200	0.11422400
C	2.45156600	-2.05895500	-1.18876300
C	2.97392300	-0.61029700	-0.98527200
H	0.88969300	-0.21803200	-0.45934400
H	0.89003000	-3.76386900	1.55135500
O	2.13779900	-2.07665300	1.32606200
C	3.24345100	-4.01959600	0.27050100
O	3.30902300	3.16206100	0.93829800
H	3.36395800	-0.25391800	-1.94494000
C	-3.55741600	-1.42734600	-0.74654200
O	-3.81069100	-2.13127300	-1.70518600
O	-4.43998700	-0.54259300	-0.22152700
C	-5.69302300	-0.45193800	-0.91797400
H	0.77272100	2.80153700	1.35542500
H	-1.72000900	2.71131400	1.67430600
H	-1.73017400	0.65292000	2.61430400
H	-3.31309000	0.56398900	1.83533700
H	-1.33220800	-3.24508500	-1.00287700
H	-4.80553000	1.91305600	1.16935800
H	-4.12645300	3.47151400	1.63542100
H	-5.04260600	3.32105900	0.12519500
H	-3.76825400	3.15924200	-1.84223000
H	-2.02148600	2.56685500	-1.83362100
H	1.51053800	-2.02751300	-1.75162000
H	3.17570100	-2.59447100	-1.81085200
H	3.10313000	-4.75043200	-0.53457600
H	4.27344000	-3.64927300	0.21746500
H	3.10254900	-4.52285600	1.23168400
H	-6.28512600	0.27430400	-0.36060900
H	-5.53585600	-0.11030700	-1.94439300
H	-6.19307200	-1.42350800	-0.93797000
O	4.09645700	-0.58578300	-0.10400700
H	3.73812000	-0.75020500	0.78526400
C	3.08617900	2.39533800	0.02186000
O	3.91192900	2.22525600	-1.02958800
C	5.17346900	2.90443900	-0.93658600
H	5.68542300	2.69897700	-1.87711100
H	5.02469500	3.97899900	-0.80348800
H	5.75103500	2.51274100	-0.09503000

TS$_{cis\text{-}12a,cis\text{-}12b}$

C	2.00347900	0.02796400	-0.86484100
C	2.28274200	1.18347900	-0.23051700
C	1.14832800	1.93413000	0.34500300
C	-0.04653500	2.03050300	-0.24859800
C	-0.80013900	-2.02360600	0.53322400
C	0.42838100	-2.73331600	0.97423500
C	-1.35398800	2.29073400	0.45356900
C	-2.38996300	1.22415300	-0.02053200
O	-0.81548000	-0.64539100	0.46574300
C	-2.08110300	-0.23989300	0.16490500
C	-2.88251600	-1.35684100	0.03613500
C	-2.04829100	-2.49753300	0.28021100
C	-1.93556700	3.69277900	0.24576900
C	-3.19694700	4.02309400	1.01082600
C	-1.34428700	4.59686100	-0.54086700
H	-0.12990700	1.74805700	-1.29953800
C	1.70192100	-2.96749600	0.22598300
C	1.99136800	-2.43997000	-1.18325900
C	2.87882800	-1.15856700	-1.18680000
H	0.95037800	-0.16506900	-1.02160800
H	0.19830400	-3.49657200	1.72296600
O	1.59889000	-1.97289600	1.27796100
C	2.52971600	-4.17334200	0.62879700
O	3.96217900	1.98404400	1.28292100

H	3.32267200	-1.05602900	-2.18529300
C	-4.31216900	-1.47273300	-0.28230500
O	-4.90919900	-2.52975000	-0.35362800
O	-4.90703000	-0.27072500	-0.49612300
C	-6.30760900	-0.33331300	-0.80991900
H	1.28860400	2.25834100	1.37603200
H	-1.20241200	2.15786200	1.53510000
H	-3.35307900	1.39970300	0.46478300
H	-2.57826200	1.39899100	-1.09008100
H	-2.37648400	-3.52701900	0.27340100
H	-4.06696900	3.46290200	0.64423000
H	-3.08975900	3.77600800	2.07600000
H	-3.43594700	5.08795600	0.93350300
H	-1.75929300	5.59411000	-0.66447700
H	-0.42185800	4.38192200	-1.07122900
H	1.06319300	-2.24123800	-1.73218100
H	2.51885000	-3.22902200	-1.72877500
H	2.31046100	-5.02881100	-0.02102300
H	3.59405700	-3.93462500	0.52773500
H	2.33099800	-4.46004900	1.66591700
H	-6.62039300	0.70137900	-0.95422600
H	-6.47159800	-0.91537200	-1.72049900
H	-6.86576700	-0.79354700	0.00967300
O	3.98794100	-1.29866300	-0.30208900
H	3.62573100	-1.23372700	0.59884200
C	3.66191100	1.59907400	0.16998400
O	4.52375000	1.53639000	-0.86407900
C	5.89507400	1.80769400	-0.53691400
H	6.43088300	1.79139700	-1.48652500
H	5.99264000	2.78300500	-0.05361100
H	6.28074300	1.03224900	0.13069500

cis-12b

C	2.08982400	0.57288000	-0.85436700
C	1.92182900	1.44676800	0.15670700
C	0.53912200	1.87008500	0.46776600
C	-0.38910500	2.06805200	-0.47660800
C	-0.33782400	-2.20814100	-0.08772600
C	0.98329100	-2.85769800	0.01539400
C	-1.87661000	1.96817800	-0.29878000
C	-2.33213500	0.66590600	-1.06802500
O	-0.55570000	-0.99665500	-0.70792200
C	-1.85367300	-0.62738500	-0.49041800
C	-2.47673600	-1.61043500	0.24989100
C	-1.49496400	-2.62272100	0.49706500
C	-2.67122100	3.18283600	-0.77472100
C	-4.17519400	3.10898100	-0.63768500
C	-2.07302400	4.28192400	-1.24519900
H	-0.06232100	2.11970900	-1.51656600
C	2.29308900	-2.69014200	-0.68004200
C	2.63023000	-1.67263400	-1.78067500
C	3.23122500	-0.33320600	-1.23831200
H	1.19542100	0.36318100	-1.42573200
H	0.83328100	-3.85250200	0.44121100
O	2.08022700	-2.15738400	0.64983400
C	3.21244500	-3.90015800	-0.68181600
O	2.85586600	1.76781600	2.34197100
H	3.79856400	0.12190000	-2.06188100
C	-3.85684200	-1.71195000	0.75172700
O	-4.28978900	-2.68469900	1.33789300
O	-4.60373100	-0.60529100	0.50599800
C	-5.95921400	-0.67024700	0.98033200
H	0.27563700	1.86332700	1.52520700
H	-2.11215200	1.80987200	0.76387700
H	-3.42056900	0.61584100	-1.08522200
H	-1.98763300	0.74854700	-2.10686100
H	-1.65379900	-3.52549700	1.06878300
H	-4.62494400	2.41619600	-1.36084700
H	-4.46465500	2.75263200	0.36001500
H	-4.63228700	4.09040800	-0.79533400
H	-2.65035200	5.15043300	-1.55255100
H	-0.99340100	4.36352000	-1.32351700
H	1.75885700	-1.45779700	-2.40828500
H	3.37767500	-2.13779900	-2.43121500
H	3.12104000	-4.45909300	-1.62060900
H	4.25012800	-3.56072100	-0.58645100
H	2.98847400	-4.57257700	0.15161300
H	-6.40898500	0.28460700	0.70660800
H	-6.49350500	-1.49788000	0.50658300
H	-5.97942100	-0.80913100	2.06421100
O	4.18463800	-0.56452500	-0.21366100
H	3.68189100	-0.91000100	0.54557600
C	2.98920600	1.82156600	1.13513600
O	4.09670000	2.27618400	0.51844200
C	5.20591400	2.56544800	1.38251000
H	5.97863200	2.98246100	0.73598000
H	4.91847100	3.28259700	2.15560900
H	5.56247500	1.64676900	1.85644300

trans-12a

C	-2.32106300	-0.25587700	0.74476700
C	-2.45687000	0.96367200	0.16767400
C	-1.26793900	1.81906100	-0.08512000
C	-0.06632500	1.67124700	0.49081400
C	0.66190300	-1.88013100	-0.90306300
C	-0.70112000	-2.44715500	-0.93980100
C	1.21372000	2.32224700	0.02343300
C	1.89499600	1.48727400	-1.14754100
O	0.61529300	-0.53091000	-1.17565500
C	1.84736200	0.00632900	-0.97428700
C	2.70904500	-1.00570700	-0.60946500
C	1.93351500	-2.22249900	-0.56583900
C	2.22691300	2.59277200	1.13401300
C	3.04251700	3.84962800	0.95184900
C	2.43002900	1.77671500	2.17305800
H	0.04256100	0.95683400	1.30287800
C	-1.41658100	-2.99070300	0.24722200
C	-2.90579200	-2.68291000	0.33680100
C	-3.23699400	-1.38690300	1.15783200
H	-1.29340700	-0.50976600	0.98726600
H	-1.32477100	-1.92064400	-1.66542500
O	-0.97710100	-3.84064500	-0.84116700
C	-0.69950500	-3.28073100	1.54851200
O	-4.73200800	0.77495900	-0.65643300
H	-2.95678400	-1.59876300	2.20150500
C	4.14652600	-0.95921600	-0.30294000
O	4.79058700	-1.91665500	0.07978300
O	4.69046600	0.26842800	-0.50010300
C	6.09089500	0.36645400	-0.19954000
H	-1.38782800	2.56366900	-0.86905300
H	0.96055200	3.28590000	-0.43858000
H	1.35836400	1.72700100	-2.07307800
H	2.93230200	1.80499500	-1.26547300
H	2.31175500	-3.20205500	-0.31457100
H	3.55615800	3.85553300	-0.01971900
H	2.39939100	4.74039200	0.96994000
H	3.79954300	3.95984400	1.73404600
H	3.18927700	1.99820200	2.91864200
H	1.86814600	0.86106400	2.32875500
H	-3.44854000	-3.50857800	0.81223600
H	-3.31338900	-2.56293900	-0.67320800
H	-0.83871100	-2.48000400	2.28355200
H	-1.09868400	-4.20362900	1.98529900
H	0.37238500	-3.41355200	1.38810500
H	6.36294600	1.40113900	-0.41116200
H	6.27664200	0.12798000	0.85109400

H	6.66857500	-0.31864400	-0.82564000
O	-4.61819000	-1.15284400	1.19721200
H	-4.86103400	-0.65860000	0.38816700
C	-3.76450900	1.46042100	-0.35198200
O	-3.77043700	2.79738000	-0.50848900
C	-4.98402400	3.35859000	-1.03898000
H	-4.80441800	4.43222400	-1.09589300
H	-5.19504000	2.94746000	-2.02941400
H	-5.82506200	3.14174700	-0.37596500

TS$_{\textit{trans}\text{-}\mathbf{12a},\textit{trans}\text{-}\mathbf{12b}}$

C	-2.23144800	-0.37165600	0.84252700
C	-2.43976800	0.81216900	0.21718600
C	-1.30627000	1.70423700	-0.12699900
C	-0.06859800	1.67965900	0.38770600
C	0.76185100	-1.85306200	-0.92980800
C	-0.61633000	-2.37545600	-1.01361600
C	1.09332700	2.41663700	-0.23061700
C	2.23636100	1.45882000	-0.80121200
O	0.78073200	-0.47988200	-1.06760300
C	2.01689700	-0.02130800	-0.74785300
C	2.82285700	-1.10422400	-0.45694600
C	2.00242600	-2.28233600	-0.58131800
C	1.72923100	3.46912900	0.67933300
C	2.68803400	4.41937500	0.00002500
C	1.44819400	3.57918300	1.98116600
H	0.15814700	1.04824700	1.24604600
C	-1.31471400	-3.05022200	0.11687200
C	-2.79977900	-2.75531400	0.28104500
C	-3.09506700	-1.55049300	1.24021400
H	-1.18931200	-0.56393200	1.08620800
H	-1.24255800	-1.76002300	-1.66235100
O	-0.90781700	-3.76970900	-1.07234600
C	-0.57895900	-3.49104000	1.36438500
O	-4.72069400	0.47360300	-0.54865200
H	-2.74333400	-1.84223400	2.23994200
C	4.23994100	-1.17095400	-0.07007200
O	4.82026200	-2.20472200	0.19954200
O	4.84784700	0.04368900	-0.04501000
C	6.23115500	0.02239100	0.34185800
H	-1.49582500	2.37670400	-0.96052900
H	0.69381800	2.95595100	-1.10007800
H	2.41834500	1.74514000	-1.84601300
H	3.17185400	1.64270500	-0.27186600
H	2.32951800	-3.29705700	-0.41208100
H	3.52892200	3.89443200	-0.47127500
H	2.18123900	4.97806700	-0.79941600
H	3.10034000	5.14366500	0.70874300
H	1.92128400	4.34170900	2.59468200
H	0.73372800	2.93376800	2.48193400
H	-3.32689700	-3.62650200	0.68839700
H	-3.23910700	-2.53147100	-0.69764500
H	-0.67386900	-2.76100200	2.17613800
H	-1.00184300	-4.43818600	1.71933000
H	0.48347700	-3.64320600	1.16783200
H	6.55913400	1.06188300	0.30781500
H	6.34308900	-0.38320600	1.35085300
H	6.81449700	-0.58834200	-0.35202500
O	-4.47323100	-1.34515100	1.39581700
H	-4.79288900	-0.88515100	0.59357800
C	-3.78192400	1.21826000	-0.29744200
O	-3.85767700	2.54420500	-0.51339600
C	-5.10790700	3.02155200	-1.04107900
H	-4.98238800	4.09887800	-1.14919500
H	-5.31809900	2.55636000	-2.00746700
H	-5.92370500	2.79446300	-0.35052600

trans-12b

C	-2.40170500	-0.04379800	0.85252900
C	-2.31654500	0.98161500	-0.03189400
C	-1.01435100	1.66653300	-0.22320000
C	-0.06748900	1.79949700	0.71513300
C	0.60123500	-2.03920900	-0.33951200
C	-0.81602700	-2.43999800	-0.51556600
C	1.38267500	2.11473100	0.44820100
C	2.27538500	0.90679800	0.92818500
O	0.71748200	-0.85301600	0.35667400
C	1.99310000	-0.39996300	0.25672000
C	2.72290500	-1.31579900	-0.47395900
C	1.81248300	-2.36733000	-0.85571800
C	1.87307700	3.41675000	1.08509800
C	3.31860600	3.78779600	0.84228400
C	1.06400600	4.21704700	1.78654200
H	-0.30621900	1.55206100	1.75028300
C	-1.69372800	-2.85410800	0.60239100
C	-3.15323500	-2.42119000	0.53262200
C	-3.45973500	-1.05541800	1.23990800
H	-1.46803700	-0.24833600	1.36787900
H	-1.30588300	-1.90212100	-1.33181500
O	-1.25166400	-3.79585600	-0.40583400
C	-1.12456200	-3.14663500	1.97571800
O	-4.43406400	0.68917300	-1.18548600
H	-3.32722700	-1.21302800	2.31953300
C	4.14158600	-1.31969300	-0.86168600
O	4.66516300	-2.20553200	-1.50851300
O	4.81975800	-0.22573200	-0.42581400
C	6.21065300	-0.19193700	-0.78381100
H	-0.78808800	1.96701500	-1.24480600
H	1.52954700	2.20540800	-0.63883100
H	3.32812100	1.13506000	0.76483300
H	2.13216400	0.79621500	2.01277100
H	2.06165100	-3.22615800	-1.46112200
H	4.01264500	3.13642900	1.38879900
H	3.57768500	3.70163600	-0.22176700
H	3.51702500	4.81662700	1.15688900
H	1.42566100	5.15031000	2.21098200
H	0.01643400	3.98409600	1.94915000
H	-3.80297200	-3.17659200	0.99060000
H	-3.44856500	-2.32913700	-0.51846600
H	-1.18290600	-2.27615000	2.63889200
H	-1.69157700	-3.96069100	2.44228400
H	-0.07932600	-3.45573500	1.90086200
H	6.59788100	0.73588100	-0.36130000
H	6.73665200	-1.05398500	-0.36538700
H	6.32881900	-0.19860200	-1.87054900
O	-4.80786000	-0.69623300	1.09253300
H	-4.91508300	-0.34714000	0.18436300
C	-3.42906700	1.34953600	-0.95241800
O	-3.19766600	2.52242900	-1.57066500
C	-4.20407800	2.94573400	-2.50683900
H	-3.84836700	3.89735500	-2.90177800
H	-4.31542200	2.21065900	-3.30800600
H	-5.16495300	3.07130500	-2.00183300

Int-13a

C	2.04773200	0.11661400	0.92963400
C	2.25673200	-1.12493900	0.41164600
C	1.13311100	-2.02354100	0.06764800
C	-0.15960700	-1.82084500	0.37572600
C	-0.38969100	1.55371400	-0.86140100
C	0.89018800	2.02659200	-0.78996700
C	-1.33349200	-2.51495100	-0.24063600
C	-1.89229100	-1.62034800	-1.50271000
O	-0.50824400	0.29807500	-1.48289300
C	-1.72323500	-0.18414400	-1.25714200
C	-2.50712500	0.79489700	-0.59350500

C	-1.66731500	1.86766100	-0.33164700
C	1.49887400	3.16929100	-0.01243200
C	2.13587600	2.60160200	1.30662600
C	-2.45369800	-2.88498900	0.72378100
H	-0.42590700	-0.99784600	1.03791800
H	1.02948300	0.31240100	1.25319900
C	0.52911200	4.30789100	0.32488800
O	2.49107400	3.73161900	-0.85739900
H	1.61910400	1.45712900	-1.36078600
O	4.66696700	-0.94796400	0.21281100
C	-3.65586900	-3.58223500	0.12932900
C	-2.34989600	-2.68339900	2.04292300
C	-3.94788900	0.64035000	-0.25342300
O	-4.60350600	-0.33221600	-0.56425900
O	-4.39941800	1.70531600	0.41276500
C	-5.80327200	1.67580800	0.77567100
H	1.39574300	-2.86450700	-0.56715400
H	-0.99987800	-3.43373100	-0.73968700
H	-1.29185500	-1.89746300	-2.37206400
H	-2.94013200	-1.85962800	-1.67468200
H	-1.93796100	2.77663300	0.18488800
H	2.78976900	3.40215700	1.66902500
H	1.35683900	2.44113000	2.06032300
H	1.09397900	5.10727500	0.81033900
H	0.08716700	4.71619800	-0.58844400
H	-0.26290300	3.98763100	1.00807600
H	3.27780400	3.15114500	-0.79996900
H	-4.31796500	-2.88238600	-0.39395000
H	-3.35613000	-4.35662200	-0.58878100
H	-4.24705300	-4.06482300	0.91186900
H	-3.13537100	-3.00999900	2.71829700
H	-1.48304200	-2.22602700	2.50890500
H	-5.98276700	2.61526500	1.29551000
H	-6.41808400	1.60220100	-0.12346300
H	-6.00090000	0.82297900	1.42803200
C	2.96539000	1.31257000	1.14221900
H	3.53015700	1.15945500	2.07722400
C	3.64448900	-1.61286600	0.10352700
O	3.85324700	1.55129800	0.06023100
H	4.44321700	0.76714200	-0.00068200
O	3.66093200	-2.89563800	-0.25897100
C	4.96334300	-3.47274000	-0.52050500
H	5.58354800	-3.41229200	0.37577800
H	4.76554100	-4.50897000	-0.78961800
H	5.45038300	-2.94033600	-1.33989300

Int-13b

C	-2.02779100	0.72145500	0.87848100
C	-1.30298000	1.64502400	0.21199100
C	0.15941900	1.72834600	0.36033700
C	0.89616300	1.27452500	1.38797900
C	-0.46813300	-1.84240700	-0.44494700
C	-1.80503200	-2.07796800	-0.31133200
C	2.35526200	0.95942900	1.26034500
C	2.50859100	-0.65326300	1.16343400
O	0.35965900	-1.55494700	0.64483300
C	1.56814200	-1.21811600	0.18639100
C	1.59792400	-1.33751400	-1.22460500
C	0.32414500	-1.71800300	-1.61359500
C	3.26521400	1.46331900	2.37238100
C	4.73169800	1.12166900	2.25646300
C	2.79479500	2.21816500	3.37122200
H	0.41445200	0.94797700	2.30954900
C	-2.67006800	-2.31799700	0.91374600
C	-3.68830300	-1.14868300	1.18545600
C	-3.40385500	0.21240700	0.53302800
H	-1.52391400	0.18378700	1.67611400
H	-2.33964400	-2.20644200	-1.24652900
O	-3.49721000	-3.43011300	0.53475400
C	-1.88607600	-2.68258900	2.18491700
O	-1.22493100	2.67917600	-1.94366300
H	-4.17375500	0.92071800	0.87155000
C	2.70861700	-1.10237100	-2.19104700
O	2.58438500	-1.31616600	-3.37455600
O	3.81385900	-0.63635500	-1.59139500
C	4.94043300	-0.38746200	-2.47302500
H	0.65961300	2.09575600	-0.53461700
H	2.72777200	1.33953800	0.30032000
H	3.53256100	-0.87785300	0.86552500
H	2.30293400	-1.07225900	2.15226700
H	-0.00893000	-1.87568900	-2.63139300
H	4.92636300	0.05339300	2.42082800
H	5.12090800	1.36892100	1.25959800
H	5.31993200	1.67499100	2.99288700
H	3.45892800	2.60892400	4.13689500
H	1.74944900	2.49816800	3.45308500
H	-3.78364800	-1.02478700	2.27036800
H	-4.65383800	-1.51319600	0.82107300
H	-2.98074100	-4.24967800	0.61916900
H	-1.30360700	-1.84467100	2.57321200
H	-2.60434300	-2.98980600	2.94981400
H	-1.19615900	-3.51513900	2.00420100
H	5.73490700	-0.02111600	-1.82500500
H	5.23543700	-1.31416900	-2.96873000
H	4.66815300	0.35918900	-3.22126200
O	-3.46006200	0.08463400	-0.89714800
H	-4.39465900	0.06320400	-1.16121600
C	-1.85343700	2.45824900	-0.92960900
O	-3.07241500	2.95041000	-0.66540000
C	-3.65884800	3.74439600	-1.71926100
H	-4.60725900	4.10136600	-1.31906600
H	-3.00510300	4.58193600	-1.97128800
H	-3.81737700	3.13015300	-2.60923400

Int-14a

C	-2.48070100	0.08993800	-0.94259700
C	-2.62516400	-1.13822800	-0.37607400
C	-1.47027600	-2.04394700	-0.17620600
C	-0.22233100	-1.83151300	-0.62572000
C	0.19390700	1.53329900	0.49690900
C	-1.07334500	2.02713100	0.57729200
C	1.02222600	-2.52028000	-0.15343200
C	1.69728200	-1.65216300	1.04891600
O	0.38449100	0.30620100	1.16277700
C	1.54724800	-0.20990400	0.80592400
C	2.24313600	0.71953200	-0.02074800
C	1.38966000	1.78669200	-0.22868500
C	-1.76653800	3.15562500	-0.15307000
C	-2.58589800	2.56726200	-1.35556900
C	2.03712500	-2.84724900	-1.24434500
H	-0.03666100	-0.99467800	-1.29755200
H	-1.51583700	0.26790000	-1.40802600
C	-0.83297700	4.26499500	-0.65088100
O	-2.62813300	3.76356900	0.79814000
H	-1.72714500	1.49293400	1.26185400
O	-4.97820100	-0.92295100	0.16658100
C	3.30270000	-3.54639400	-0.80103400
C	1.80137900	-2.60035700	-2.53861200
C	3.63198300	0.54550600	-0.51712700
O	4.42024900	-0.25105500	-0.03899400
O	3.89930200	1.38243100	-1.51845000
C	5.24663000	1.32898300	-2.05307300
H	-1.65816400	-2.89251400	0.47462100
H	0.75647600	-3.45865900	0.35081200
H	1.16893400	-1.89720200	1.97201300
H	2.74475300	-1.92223200	1.15435600

H	1.59443600	2.66021100	-0.82984100
H	-3.27235600	3.36946500	-1.64703500
H	-1.92082100	2.37457100	-2.20495700
H	-1.44414600	5.06198600	-1.08121700
H	-0.26598000	4.68928300	0.18264200
H	-0.14434500	3.90985600	-1.42324300
H	-3.41890200	3.19070300	0.87135300
H	4.00681600	-2.85828000	-0.31729200
H	3.08645900	-4.35040500	-0.08573000
H	3.81851800	-3.99079500	-1.65622900
H	2.51854600	-2.89280400	-3.30037700
H	0.88707000	-2.14068500	-2.89955400
H	5.26971300	2.08018100	-2.84052500
H	5.96890200	1.56186000	-1.26820100
H	5.44653500	0.33402700	-2.45513000
C	-3.40296200	1.29620600	-1.04681700
H	-4.09789600	1.13531200	-1.88814200
C	-3.95910100	-1.60163100	0.13485100
O	-4.12486600	1.57075500	0.14511000
H	-4.70455400	0.79380500	0.30923500
O	-3.94156200	-2.88059100	0.51334400
C	-5.20037100	-3.43228300	0.96791500
H	-5.94328000	-3.37173500	0.17030500
H	-4.98170100	-4.46871400	1.21987600
H	-5.55609000	-2.88277800	1.84180800
C	3.96964300	0.65165200	3.90170300
H	3.11703600	0.76727000	4.57491000
H	4.37221400	1.64806200	3.67217500
H	4.74243800	0.06689400	4.41765600
O	3.49129900	-0.01794000	2.73598400
H	4.22886500	-0.14342600	2.11638900

Int-14b

C	-2.44146700	-1.34403200	1.24589000
C	-1.71777500	-0.31953300	1.73428900
C	-0.26428100	-0.42178500	1.99645400
C	0.52269900	-1.43089800	1.58826800
C	-0.91953100	0.33482000	-1.49135700
C	-2.23088300	0.21014300	-1.82186800
C	1.98612000	-1.28398100	1.29832300
C	2.09330200	-1.23634200	-0.32127300
O	-0.06334900	-0.74874100	-1.26896000
C	1.11774400	-0.27429900	-0.84995700
C	1.09429000	1.14325000	-0.85176700
C	-0.18379900	1.51916100	-1.22454300
C	2.92502600	-2.36499300	1.80877100
C	4.39476800	-2.02381900	1.77529000
C	2.47187400	-3.53018400	2.28534300
H	0.06719100	-2.33234900	1.18162400
C	-3.06080600	-0.99980900	-2.16777000
C	-3.27021700	-1.99923800	-0.99443800
C	-3.65958400	-1.31260300	0.36585600
H	-2.00351700	-2.33886300	1.30354000
H	-2.75341800	1.15546300	-1.93025600
O	-4.33936600	-0.42720600	-2.51480500
C	-2.45728800	-1.71913100	-3.39307800
O	-1.67608400	2.02232200	1.19461600
H	-4.46945700	-1.90251000	0.82319600
C	2.21095700	2.03595500	-0.44962200
O	3.25734300	1.64467400	0.03433300
O	1.90357100	3.31260200	-0.67889300
C	2.90285500	4.29025900	-0.29992600
H	0.20265100	0.48999300	2.36357100
H	2.34085000	-0.30857000	1.64950900
H	3.11139200	-0.95807000	-0.61271300
H	1.86084800	-2.23404300	-0.70258100
H	-0.57232700	2.52475100	-1.29194100
H	4.72664300	-1.70424600	0.77985500
H	4.60866700	-1.19089700	2.45940300
H	5.00423800	-2.87708800	2.08506000
H	3.15741200	-4.28563400	2.65853600
H	1.41522300	-3.77124100	2.34876400
H	-2.37593300	-2.61766000	-0.87211100
H	-4.06981800	-2.67450700	-1.32315400
H	-4.85465100	-1.09723300	-2.99356400
H	-1.45287700	-2.09380200	-3.17961000
H	-3.08779900	-2.57454000	-3.66239600
H	-2.41073200	-1.03910500	-4.24830900
H	2.45712800	5.25623800	-0.53048900
H	3.12298000	4.20526100	0.76598300
H	3.81499800	4.13458200	-0.87974100
O	-4.08690200	0.02425900	0.15796100
H	-4.71186400	0.02445300	-0.59142200
C	-2.22517200	1.09864900	1.77507800
O	-3.27458900	1.23255600	2.58272800
C	-3.85063200	2.55253300	2.66133800
H	-4.64186700	2.47736300	3.40601900
H	-3.09645800	3.28096100	2.96691400
H	-4.26172700	2.83456200	1.68898200
C	6.30605600	-0.32827700	-1.79624400
H	6.36340100	-1.24903400	-2.38135600
H	7.04914800	-0.38187200	-0.98920100
H	6.55475500	0.51659800	-2.45318200
O	4.97382800	-0.23713400	-1.29614600
H	4.88377600	0.56928800	-0.76395200

TS1a

C	-2.53083600	0.13310000	-1.29636200
C	-2.58852100	-1.10896600	-0.75282900
C	-1.39282400	-1.98808100	-0.75347000
C	-0.14393800	-1.51563300	-0.87603700
C	0.16760600	1.32759700	0.68461000
C	-1.09027800	1.81567900	0.69984800
C	1.13416800	-2.25318500	-0.59188900
C	1.66601900	-1.83028500	0.82832800
O	0.42957800	0.15364300	1.43610200
C	1.69434700	-0.32821700	1.15534100
C	2.29010800	0.67936700	0.19867000
C	1.36823800	1.64528200	-0.04192200
C	-1.73451100	3.02550900	0.02925100
C	-2.50835900	2.63709300	-1.27118200
C	2.20715500	-2.14134000	-1.67832400
H	-0.01352600	-0.45724600	-1.07167900
H	-1.65908200	0.31157000	-1.92235800
C	-0.75873800	4.16697900	-0.28404900
O	-2.63715700	3.56032400	0.99262100
H	-1.78490800	1.26311500	1.32625600
O	-4.89412200	-1.03240800	-0.01119400
C	3.46780100	-2.95085800	-1.46226100
C	2.02775400	-1.43701800	-2.80142500
C	3.72642900	0.68071900	0.00153100
O	4.45370000	0.10666700	0.86509200
O	4.21765700	1.31093500	-1.02520200
C	5.67017300	1.39751300	-1.14627500
H	-1.55748400	-3.03639100	-0.52514400
H	0.91000700	-3.32416200	-0.48285000
H	1.01376100	-2.28840900	1.57852100
H	2.66972300	-2.24125800	0.97090000
H	1.50971100	2.52816500	-0.64886900
H	-3.15044900	3.49677700	-1.49065900
H	-1.81051200	2.52893600	-2.10948600
H	-1.33613900	5.03349900	-0.61610400
H	-0.20579600	4.45555300	0.61456200
H	-0.05569300	3.90684400	-1.08159000
H	-3.42551600	2.98133500	0.98025500
H	4.09264700	-2.55918700	-0.64799300

H	3.23124500	-3.99127500	-1.20334400
H	4.08265000	-2.96498900	-2.36628200
H	2.78498900	-1.41915800	-3.58054800
H	1.11706400	-0.88418300	-3.00779500
H	5.83600100	2.01361900	-2.02719400
H	6.08616800	1.86243500	-0.25134800
H	6.08201800	0.39659000	-1.28193300
C	-3.39596200	1.37248400	-1.18542800
H	-4.06472500	1.39169000	-2.06506400
C	-3.82340900	-1.62387000	-0.08397500
O	-4.14620200	1.49139500	0.00975200
H	-4.69808600	0.68192200	0.09280400
O	-3.66832600	-2.86512100	0.39416000
C	-4.84170300	-3.46866300	0.98314500
H	-5.64331500	-3.53205200	0.24433800
H	-4.52428600	-4.46177900	1.29842400
H	-5.18120500	-2.87784500	1.83650400
C	2.39779600	-0.92131900	3.58579300
H	1.34778900	-0.82049000	3.86213400
H	3.03048600	-0.46845600	4.34948200
H	2.66759200	-1.96829900	3.43269000
O	2.61161000	-0.13973600	2.37902600
H	3.66387700	-0.12578400	1.82398900

TS1b

C	2.54465400	-0.90805800	1.38086400
C	1.89423300	-1.59216700	0.42223100
C	0.46634100	-1.94993400	0.55114600
C	-0.37822500	-1.33690100	1.39650100
C	0.32068600	1.21506400	-0.48346800
C	1.63002500	1.52091500	-0.49780600
C	-1.87805500	-1.39792000	1.34039000
C	-2.44060700	0.06619300	1.30275100
O	-0.57730800	1.46645400	0.57215400
C	-1.83739000	0.98222500	0.23071500
C	-1.70249900	0.45414800	-1.17335400
C	-0.40109900	0.55088800	-1.53930900
C	-2.54810000	-2.17023800	2.48177600
C	-4.05052300	-2.31692800	2.40585500
C	-1.83912900	-2.72108500	3.47108900
H	0.01941000	-0.61941900	2.11071400
C	2.51526100	2.30067100	0.45179500
C	3.13018500	1.47124700	1.62049200
C	3.68939500	0.05918100	1.24947300
H	2.08130000	-0.86920300	2.36616800
H	2.14889100	1.15246000	-1.37603500
O	3.60707600	2.70487300	-0.41797300
C	1.81257600	3.55035400	1.00218500
O	1.88916500	-1.40740400	-1.97354300
H	4.45435300	-0.18684700	2.00487900
C	-2.90618700	0.25307500	-1.95538800
O	-3.96465900	0.84538400	-1.60381300
O	-2.83322900	-0.49921600	-3.01942000
C	-4.01580800	-0.59743100	-3.86653700
H	0.06659900	-2.62924400	-0.19977400
H	-2.17297000	-1.88704600	0.39919700
H	-3.52115600	0.02005000	1.13604300
H	-2.27457400	0.53080600	2.28147100
H	0.05343800	0.24638200	-2.47180800
H	-4.57632000	-1.36446200	2.55698400
H	-4.36452900	-2.70202800	1.42584100
H	-4.41374600	-3.01040600	3.16881900
H	-2.32439600	-3.28531500	4.26258100
H	-0.75713900	-2.65050800	3.52121800
H	2.39984600	1.37381500	2.43095500
H	3.94676100	2.08845800	2.01900900
H	4.11489000	3.39500600	0.04049600
H	0.93270800	3.28121500	1.59232500
H	2.49446600	4.11282300	1.65134000
H	1.50173300	4.19930400	0.17819900
H	-3.70491600	-1.22129600	-4.70169900
H	-4.82779900	-1.06434300	-3.30713300
H	-4.31124500	0.39754100	-4.20305300
O	4.26513300	0.02857900	-0.04369500
H	4.43489900	0.94740300	-0.32447900
C	2.44834800	-1.80297000	-0.96095300
O	3.55068800	-2.54897300	-0.95237100
C	4.19012100	-2.74955300	-2.22825100
H	5.01882600	-3.42862500	-2.03172900
H	3.48995400	-3.18581400	-2.94415000
H	4.55859700	-1.79362600	-2.60812300
C	-3.28729700	2.93546600	1.17532100
H	-2.41987400	3.25513700	1.75260600
H	-3.96629200	2.33362300	1.78210200
H	-3.80297500	3.80166200	0.76041700
O	-2.79540000	2.18123200	0.03087900
H	-3.55191100	1.69762300	-0.67623500

Int-23a

C	-2.48870900	0.15275900	-1.32403400
C	-2.55340100	-1.09403500	-0.79115500
C	-1.35772000	-1.97140700	-0.77897100
C	-0.10613400	-1.50087600	-0.88305500
C	0.15829700	1.27679800	0.73987200
C	-1.10232900	1.78182800	0.72512500
C	1.16061800	-2.24676700	-0.57266200
C	1.64870400	-1.85330800	0.86778700
O	0.41127400	0.13409800	1.50595800
C	1.72268300	-0.35897000	1.26633200
C	2.29130200	0.65692700	0.28332500
C	1.34460500	1.60287600	0.01180800
C	-1.71876600	3.00633500	0.05765200
C	-2.46509200	2.65158800	-1.26835400
C	2.26465700	-2.13096900	-1.62749600
H	0.03079300	-0.44281400	-1.07531100
H	-1.60672100	0.33623700	-1.93408900
C	-0.72970100	4.14961900	-0.20831800
O	-2.63990700	3.52795500	1.01135000
H	-1.81351000	1.23703100	1.33951900
O	-4.87356500	-1.02899900	-0.09399100
C	3.48470200	-3.00072600	-1.41304700
C	2.14841600	-1.37404600	-2.72514100
C	3.67817300	0.69581200	-0.02190400
O	4.53701700	0.05233200	0.72022300
O	4.11701600	1.39097100	-1.02020700
C	5.55895900	1.50644000	-1.24179200
H	-1.52529900	-3.01984900	-0.55377500
H	0.92538500	-3.31788700	-0.49096900
H	0.95969100	-2.31143600	1.58414800
H	2.63313000	-2.29811800	1.04580400
H	1.47069400	2.47008000	-0.62052800
H	-3.09794300	3.51927700	-1.48329300
H	-1.74930400	2.55695600	-2.09300600
H	-1.29755400	5.02774400	-0.52581500
H	-0.18965000	4.41041300	0.70633800
H	-0.01563700	3.90791800	-1.00172900
H	-3.43378200	2.95760800	0.96576500
H	4.08521900	-2.68803900	-0.54830000
H	3.19725600	-4.04456400	-1.23175500
H	4.13863600	-2.98302000	-2.28933500
H	2.92809500	-1.35824200	-3.48214200
H	1.26660200	-0.77791200	-2.93667000
H	5.64916700	2.19798700	-2.07616300
H	6.03702800	1.90036100	-0.34403200
H	5.96203100	0.52565200	-1.49685000
C	-3.35780500	1.38964100	-1.22453900

H	-3.99950700	1.42012800	-2.12393300
C	-3.79985100	-1.61708600	-0.14900700
O	-4.14313000	1.49789400	-0.05154500
H	-4.68932700	0.68272200	0.01454800
O	-3.65015300	-2.85993600	0.32469100
C	-4.83189700	-3.47029800	0.88976600
H	-5.61997700	-3.53236300	0.13643400
H	-4.51663800	-4.46387100	1.20562700
H	-5.18805300	-2.88479900	1.73996100
C	2.15613800	-0.87614000	3.64453600
H	1.11118000	-0.64159900	3.85918700
H	2.79330400	-0.46793800	4.42974800
H	2.29971300	-1.95886800	3.57827600
O	2.56213400	-0.21099700	2.42938400
H	4.05439300	-0.24130900	1.55507000

Int-23b

C	-2.51819700	-0.63703900	-1.49601800
C	-1.87790400	-1.48378300	-0.66649600
C	-0.44997400	-1.81162500	-0.84235500
C	0.39880500	-1.04051100	-1.54516100
C	-0.25928500	1.13835600	0.60547600
C	-1.58661900	1.39448700	0.68428800
C	1.89845400	-1.11263200	-1.52511000
C	2.46891900	0.32631800	-1.29982300
O	0.63347500	1.63874900	-0.33109100
C	1.95267400	1.11680000	-0.07465600
C	1.76494200	0.30942300	1.19980500
C	0.43394700	0.27563500	1.51251300
C	2.53431000	-1.72918600	-2.77713700
C	4.03435000	-1.91222900	-2.74791400
C	1.80285400	-2.11753400	-3.82552200
H	-0.00234200	-0.19663000	-2.10113400
C	-2.49261500	2.34195100	-0.06825800
C	-3.14740000	1.73688100	-1.34995900
C	-3.68080200	0.27590600	-1.21965300
H	-2.04224200	-0.42473900	-2.45258000
H	-2.09196700	0.82951400	1.45968300
O	-3.55676700	2.57154700	0.89545400
C	-1.80517900	3.67371700	-0.40084000
O	-1.90870700	-1.72938500	1.72214000
H	-4.43396600	0.14292300	-2.01449200
C	2.86260900	-0.12033600	1.98045200
O	4.05848400	0.37852100	1.79373000
O	2.70427300	-1.00293200	2.91701400
C	3.82249900	-1.32938700	3.79957500
H	-0.05630000	-2.62785800	-0.23909700
H	2.20637900	-1.73580400	-0.67050000
H	3.55765900	0.26173600	-1.20103300
H	2.26348200	0.92446400	-2.19486200
H	-0.04877400	-0.24958300	2.32588100
H	4.57585500	-0.95728700	-2.77323600
H	4.35454600	-2.43652500	-1.83677100
H	4.37227500	-2.49856700	-3.60644700
H	2.26773300	-2.56876900	-4.69762700
H	0.72218700	-2.01905700	-3.85128600
H	-2.44546200	1.79527100	-2.18874400
H	-3.98158800	2.40603200	-1.60059600
H	-4.07088300	3.33988600	0.59584800
H	-0.94412200	3.52103100	-1.05621600
H	-2.50696800	4.34245900	-0.91358400
H	-1.46716700	4.16168200	0.51799900
H	3.40205800	-2.02691700	4.52029900
H	4.61915700	-1.79600500	3.21866500
H	4.18161000	-0.42229500	4.28775100
O	-4.27127700	0.02306700	0.04213000
H	-4.40132600	0.87713400	0.49613800
C	-2.45176400	-1.94217500	0.64730400
O	-3.54814800	-2.67813700	0.48962400
C	-4.20653200	-3.10441800	1.69945200
H	-5.02832500	-3.73955200	1.37142800
H	-3.51503200	-3.65948000	2.33716400
H	-4.58553900	-2.23183800	2.23664400
C	3.13293900	3.17275000	-0.73181600
H	2.19600500	3.58693600	-1.11150600
H	3.73019900	2.76647200	-1.55364700
H	3.69667300	3.95219300	-0.21826700
O	2.85428100	2.17195800	0.26886100
H	3.96509400	1.16785700	1.18236100

15a

	-2.34961900	0.11743600	-1.35295300
C	-2.46791800	-1.12164900	-0.80940100
C	-1.28110600	-2.00969900	-0.71123600
C	-0.02670100	-1.53829900	-0.71862800
C	0.17348300	1.27525200	0.83957700
C	-1.08173100	1.76964500	0.82874500
C	1.23419400	-2.28315800	-0.38873000
C	1.77171100	-1.82076800	1.01605400
O	0.42884700	0.12527400	1.57497200
C	1.79193700	-0.31665600	1.38304900
C	2.33132900	0.68308800	0.37404500
C	1.39169400	1.61705000	0.12635200
C	-1.70674600	2.97486400	0.13190000
C	-2.28337400	2.62665300	-1.27629000
C	2.29076500	-2.24941700	-1.49679100
H	0.11893600	-0.47732700	-0.87693100
H	-1.41848900	0.27876800	-1.89101400
C	-0.76018500	4.17603600	0.00110400
O	-2.76999000	3.44402800	0.96659800
H	-1.79024300	1.21891700	1.44161100
O	-4.83935900	-1.05918100	-0.30102200
C	3.54405700	-3.05628600	-1.24660800
C	2.10510800	-1.60811800	-2.65608200
C	3.71231400	0.64120400	-0.13159200
O	4.52266700	-0.22849300	0.12817200
O	3.98720100	1.69879600	-0.93178500
C	5.31721600	1.72552700	-1.47107000
H	-1.46726700	-3.06116600	-0.51557100
H	0.98520500	-3.34656800	-0.24891600
H	1.13777000	-2.30035000	1.76862600
H	2.78959300	-2.19201600	1.15775800
H	1.51521400	2.47323600	-0.52038200
H	-2.87878200	3.49372800	-1.58418500
H	-1.46647000	2.51775200	-2.00019400
H	-1.32619900	5.03203700	-0.37838600
H	-0.35514800	4.44053000	0.98191600
H	0.06861100	3.98238000	-0.68558800
H	-3.50578000	2.81499500	0.83397900
H	4.19279000	-2.56936700	-0.51050800
H	3.30177200	-4.05808700	-0.86634300
H	4.12099600	-3.17597700	-2.16884000
H	2.85447100	-1.64354300	-3.44297300
H	1.20568300	-1.04123000	-2.87466100
H	5.36911900	2.63259200	-2.07410200
H	6.05779300	1.75490600	-0.66743300
H	5.49859600	0.84191000	-2.08904700
C	-3.19212100	1.37302900	-1.36632700
H	-3.67264900	1.41571000	-2.36377500
C	-3.74737500	-1.61771800	-0.23467000
O	-4.17119800	1.49455100	-0.35148800
H	-4.66397700	0.64484400	-0.31135300
O	-3.62253100	-2.82596900	0.34280900
C	-4.83210700	-3.39691100	0.87067800
H	-5.57057900	-3.53069500	0.07625800
H	-4.53691200	-4.35880500	1.28992800

H	-5.25293700	-2.75037100	1.64451700
C	2.09075100	-0.75379900	3.74521900
H	1.01500200	-0.61510600	3.90023800
H	2.63559800	-0.28486000	4.56797500
H	2.32601300	-1.82614300	3.73551800
O	2.53421600	-0.09332300	2.56346900

15b

C	2.36652600	-0.67209500	1.58730600
C	1.72134100	-1.51639700	0.76396700
C	0.26760300	-1.76579900	0.87834300
C	-0.56792200	-0.98337100	1.57874900
C	0.32366700	1.18816200	-0.74003400
C	1.64782300	1.40292800	-0.84649100
C	-2.06444300	-0.94211700	1.45857400
C	-2.50326500	0.53370800	1.19948200
O	-0.49831200	1.62906600	0.27476400
C	-1.88585700	1.25159300	-0.01802600
C	-1.75596400	0.42842100	-1.28557600
C	-0.47027900	0.41590400	-1.67842400
C	-2.82228600	-1.51149000	2.66127800
C	-4.33153100	-1.48175500	2.57691900
C	-2.19503600	-2.04196300	3.71577000
H	-0.15205500	-0.18929900	2.19409200
C	2.58870300	2.26155900	-0.02825400
C	3.08073500	1.67466500	1.32774400
C	3.57341200	0.19001000	1.32725600
H	1.84715900	-0.37734900	2.49881400
H	2.12175400	0.89497500	-1.67873600
O	3.75980100	2.36571100	-0.89769800
C	2.01053900	3.66634700	0.19679500
O	1.89786500	-1.89706100	-1.60904900
H	4.26304200	0.09187100	2.18354900
C	-2.90750200	-0.22210700	-1.92621200
O	-3.99784500	-0.37938900	-1.40423600
O	-2.61216100	-0.65602200	-3.17323400
C	-3.67396800	-1.34665400	-3.84840900
H	-0.14364300	-2.52404400	0.21443300
H	-2.37001200	-1.53484000	0.58454800
H	-3.58255600	0.55729400	1.03415900
H	-2.28236500	1.12292800	2.09763200
H	-0.05289700	-0.09021800	-2.53773200
H	-4.73028700	-0.46526100	2.68869500
H	-4.68174100	-1.84709300	1.60277300
H	-4.78064200	-2.10035600	3.35990900
H	-2.75213500	-2.46301400	4.54921900
H	-1.11232800	-2.09151100	3.77943300
H	2.29206500	1.79119500	2.07883700
H	3.90915900	2.32082000	1.65410400
H	4.35594400	3.02107000	-0.49867600
H	1.06842600	3.61143000	0.74845600
H	2.71084300	4.29004300	0.76869100
H	1.82087500	4.14406200	-0.76870000
H	-3.26876900	-1.62097500	-4.82280400
H	-3.97011700	-2.23927300	-3.29033700
H	-4.54441500	-0.69514400	-3.96272300
O	4.27065400	-0.15447800	0.14267900
H	4.32673200	0.64653900	-0.41433400
C	2.34807200	-2.05793000	-0.48863100
O	3.41223200	-2.83309600	-0.21713000
C	4.12832500	-3.31429700	-1.36278900
H	4.91129700	-3.96223800	-0.96730500
H	3.46607500	-3.87102400	-2.03102000
H	4.56820000	-2.47352200	-1.90610500
C	-2.81405900	3.38089300	0.64857100
H	-1.87369200	3.65249900	1.14294200
H	-3.53835500	3.04570500	1.40190600
H	-3.21800400	4.26026900	0.14156200
O	-2.60665400	2.40841600	-0.37019000

TS2a

C	-2.19046500	0.35138800	-0.97127500
C	-2.32124300	-1.03102100	-0.63268300
C	-1.12558400	-1.83841400	-0.58108900
C	0.12015400	-1.45554600	-0.95566500
C	-0.00311500	1.00948200	0.61127800
C	-1.35658400	1.38033700	0.48248600
C	1.37023900	-2.16929800	-0.51011400
C	1.78215100	-1.76248000	0.96422000
O	0.33345500	0.10358300	1.56314600
C	1.74434300	-0.28231600	1.40395200
C	2.24354000	0.71575500	0.38110800
C	1.18668700	1.42013600	-0.08927100
C	-1.86888900	2.82337500	0.10489200
C	-2.55026600	2.72299400	-1.28017600
C	2.52652600	-2.13314100	-1.51131100
H	0.26497200	-0.53173100	-1.50220800
H	-1.38160600	0.50430000	-1.68292700
C	-0.81812900	3.93396900	0.15176200
O	-2.82490900	3.18533100	1.09405200
H	-1.93915700	1.00055400	1.31790100
O	-4.67798000	-1.08542200	-0.07802700
C	3.78707200	-2.86493800	-1.10616400
C	2.42959700	-1.57206900	-2.72250500
C	3.64650200	0.79563400	-0.03730100
O	4.52756400	0.04175500	0.33608800
O	3.85418600	1.82062300	-0.89840500
C	5.20229400	1.95251800	-1.37426900
H	-1.23155100	-2.80524300	-0.09608900
H	1.12034900	-3.23687400	-0.40396600
H	1.11371400	-2.30295500	1.64052100
H	2.80070500	-2.10658800	1.15790300
H	1.22333300	2.16064700	-0.87453800
H	-3.24278600	3.55858800	-1.41869400
H	-1.80673800	2.75717500	-2.08665300
H	-1.32695100	4.88980600	-0.00687600
H	-0.33234000	3.96673300	1.13131500
H	-0.05422400	3.82435100	-0.62233400
H	-3.61481600	2.63779800	0.89931800
H	4.36323600	-2.29453800	-0.36902300
H	3.55495600	-3.84257100	-0.66189000
H	4.43386900	-3.03478500	-1.97226500
H	3.25393600	-1.62683200	-3.42917800
H	1.53788900	-1.06214400	-3.07023200
H	5.19601600	2.82408200	-2.02948900
H	5.89175600	2.10323100	-0.53946900
H	5.50275400	1.05813900	-1.92695600
C	-3.27785700	1.37940400	-1.30392900
H	-3.66482300	1.15086800	-2.31197700
C	-3.57386100	-1.63331500	-0.18231300
O	-4.34169300	1.48058700	-0.37389900
H	-4.66599900	0.56005400	-0.20984700
O	-3.46084000	-2.95777100	0.08904300
C	-4.66922600	-3.61689900	0.48814800
H	-5.42691900	-3.54581000	-0.29677400
H	-4.38814700	-4.65752900	0.65540600
H	-5.06867300	-3.17514200	1.40509500
C	1.99354900	-0.81299500	3.74148500
H	0.90948100	-0.75731200	3.88929600
H	2.49757000	-0.36022800	4.59768800
H	2.30312100	-1.86227400	3.66361100
O	2.40427500	-0.04732700	2.60871700

TS2b

C	2.36418500	0.04834200	-0.72978900
C	2.09882900	1.32257600	-0.18363800

C	0.77010000	1.83450700	-0.25010800
C	-0.21704200	1.24664800	-0.99358800
C	-0.07662800	-1.13756500	0.21569600
C	1.31589200	-1.34413400	0.26985200
C	-1.69197500	1.53395100	-0.96191300
C	-2.48201200	0.25753500	-1.40257600
O	-0.88465900	-1.52504200	-0.81755800
C	-2.24800000	-1.00830100	-0.54433400
C	-2.18917200	-0.67053000	0.92971700
C	-0.88500500	-0.65906800	1.30110400
C	-2.14431000	2.73021100	-1.80735400
C	-3.49600800	3.30532600	-1.45562800
C	-1.39828000	3.24093300	-2.79174400
H	0.07021600	0.50969500	-1.73502700
C	2.07500400	-2.58716400	-0.26032800
C	3.24344200	-2.13286000	-1.17369800
C	3.66973200	-0.74065200	-0.69209600
H	1.82940900	-0.15780200	-1.65665900
H	1.68350700	-1.10033000	1.26227600
O	2.65070800	-3.12705000	0.96647500
C	1.20243200	-3.65560400	-0.91721700
O	2.72453500	2.74933200	1.64242700
H	4.40316400	-0.28568500	-1.36771200
C	-3.36167800	-0.24272900	1.69240700
O	-4.45064200	0.02056100	1.21128800
O	-3.09076300	-0.15056200	3.01972200
C	-4.18085300	0.30661400	3.83263400
H	0.53707800	2.66730400	0.41120300
H	-1.97925200	1.75628300	0.07724400
H	-3.55513300	0.45778500	-1.33788600
H	-2.23291100	0.04353300	-2.44816800
H	-0.48776900	-0.36095500	2.26267800
H	-4.29358900	2.55354600	-1.50434300
H	-3.49826200	3.69086800	-0.42684100
H	-3.76523800	4.12674800	-2.12646200
H	-1.74830600	4.08176400	-3.38532400
H	-0.41181100	2.85494500	-3.02993700
H	2.91438600	-2.07831000	-2.21839200
H	4.06764400	-2.85503800	-1.12919500
H	3.11969100	-3.94500700	0.73002100
H	0.68733900	-3.26469900	-1.79787900
H	1.82944400	-4.49910600	-1.23590100
H	0.44997200	-4.02441500	-0.21504200
H	-3.79947700	0.31419300	4.85430000
H	-4.49350700	1.31094700	3.53276400
H	-5.03570500	-0.36957700	3.74637500
O	4.27476700	-0.79726500	0.59388700
H	3.90147700	-1.57437100	1.05078600
C	3.04633100	2.06397300	0.68709600
O	4.32139000	1.96393600	0.24838100
C	5.31167500	2.55745300	1.09637000
H	6.26409700	2.40087400	0.58852400
H	5.11716900	3.62475800	1.23208400
H	5.31839500	2.06660200	2.07352000
C	-3.35040700	-2.44483900	-2.12156900
H	-2.39858500	-2.69664300	-2.60409100
H	-3.87323500	-1.68534900	-2.71543300
H	-3.97317900	-3.34025900	-2.06875600
O	-3.15840300	-2.03016600	-0.77143600

TS3a

C	-1.59961000	-0.11332900	0.97914000
C	-1.87601200	1.07736100	0.29971400
C	-0.87593700	2.01885400	-0.12912800
C	0.44271400	1.94421400	0.20021000
C	0.26747800	-1.32706400	-0.74280500
C	-1.13270800	-1.48184300	-0.62932200
C	1.57835900	2.58079500	-0.52773800
C	2.31648300	1.48236600	-1.49573400
O	0.62462000	-0.16098700	-1.41016600
C	1.92829900	0.08773900	-1.20415100
C	2.48618300	-0.98136400	-0.50009500
C	1.42752000	-1.86575000	-0.19470500
C	-1.87202200	-2.69332900	-0.05674200
C	-2.12846800	-2.45945000	1.45025800
C	2.52273000	3.27993800	0.45714800
H	0.74887400	1.26726800	0.99748500
H	-0.61995900	-0.18447200	1.44517700
C	-1.17743200	-4.03216100	-0.34481700
O	-3.13300600	-2.65181900	-0.76110500
H	-1.70880300	-0.95561500	-1.38640300
O	-3.54038300	0.72851800	-1.35848500
C	2.03117800	4.63870200	0.90027500
C	3.65426800	2.74246800	0.91973000
C	3.91539500	-1.11371800	-0.12842500
O	4.75325000	-0.26105700	-0.35035900
O	4.15261600	-2.28293600	0.47858000
C	5.52531500	-2.51877900	0.87467000
H	-1.18796200	2.74898000	-0.87359900
H	1.17722900	3.33421700	-1.21472000
H	1.99131600	1.73397800	-2.50908800
H	3.39537600	1.60479200	-1.42358100
H	1.52077200	-2.77582200	0.37823000
H	-2.85243200	-3.18601900	1.83495500
H	-1.19523500	-2.59553500	2.01146700
H	-1.81498700	-4.85104400	0.00862900
H	-1.01288100	-4.16051500	-1.41798800
H	-0.22153200	-4.12291700	0.17606500
H	-3.60005900	-3.48941700	-0.60239700
H	1.94719800	5.32614600	0.04783100
H	1.03466100	4.57995500	1.35821800
H	2.71214600	5.08641500	1.62846100
H	4.26441100	3.28620200	1.63568800
H	4.03563000	1.77507400	0.60890400
H	5.52477600	-3.50591300	1.33424200
H	6.17612600	-2.49666000	-0.00170400
H	5.84616300	-1.75614500	1.58740800
C	-2.63733200	-1.01802600	1.63497400
H	-2.66149600	-0.77247300	2.70493400
C	-3.24005600	1.28537000	-0.32160300
O	-3.94505500	-0.81640400	1.15419500
H	-4.01678100	-1.29320100	0.30267900
O	-3.95114400	2.19072300	0.33177900
C	-5.28446800	2.44836000	-0.18078000
H	-5.23445400	2.74411100	-1.23022400
H	-5.89142200	1.54719900	-0.07357100
H	-5.67512200	3.25498600	0.43693900

TS3b

C	-2.07128900	0.59388600	0.75107000
C	-1.39213600	1.35885400	-0.18874300
C	0.03286000	1.58305600	-0.10899800
C	0.79629300	1.46694200	1.01161700
C	-0.34130600	-1.58382600	-0.03553300
C	-1.72829000	-1.42794200	0.15860500
C	2.27846600	1.30578000	0.99820600
C	2.63074500	-0.25261900	1.31972300
O	0.51935100	-1.27469200	1.01247600
C	1.76232700	-1.12226200	0.50664700
C	1.76037900	-1.46258000	-0.84421100
C	0.41488900	-1.71145300	-1.19136700
C	3.04844300	2.19976200	1.96734500
C	4.54361300	1.99481800	2.02058900
C	2.44255100	3.14871500	2.68856000
H	0.31670100	1.36074200	1.98319800
C	-2.59706800	-1.97745900	1.31868900

C	-3.72239800	-0.95551200	1.65259900
C	-3.55877600	0.33121500	0.82579700
H	-1.55713500	0.45560500	1.70013300
H	-2.28946800	-1.43302800	-0.77247200
O	-3.23710400	-3.11916800	0.74764300
C	-1.80840700	-2.38408600	2.56687800
O	-1.65103600	1.05894200	-2.51620700
H	-4.06086900	1.17409100	1.32521700
C	2.87459800	-1.49155600	-1.82464000
O	2.72569600	-1.84050600	-2.97410200
O	4.03970200	-1.08362900	-1.28648100
C	5.17654800	-1.11040800	-2.18357900
H	0.52545800	1.78695700	-1.05767600
H	2.65532800	1.47593400	-0.01888500
H	3.67999100	-0.40422000	1.07220500
H	2.46752000	-0.42771000	2.38645700
H	0.04735900	-1.93276700	-2.18413900
H	4.81745800	1.03964100	2.48620400
H	4.98272600	1.99938200	1.01400600
H	5.02183200	2.78957000	2.59856900
H	3.01165900	3.79390600	3.35143000
H	1.37475800	3.34014000	2.63973400
H	-3.69653300	-0.71023100	2.71996800
H	-4.69029000	-1.42366600	1.45288200
H	-2.60451500	-3.85730200	0.72641600
H	-1.30640300	-1.53231200	3.03505200
H	-2.50276400	-2.81617700	3.29355100
H	-1.04323700	-3.13252000	2.33252200
H	6.02055300	-0.76351300	-1.58897300
H	5.34559400	-2.12769400	-2.54223600
H	4.99963300	-0.44965300	-3.03475800
O	-4.03711400	0.18335000	-0.50510300
H	-5.00444300	0.11268100	-0.48308900
C	-2.00132200	1.67744900	-1.53325300
O	-2.84900400	2.69570100	-1.49035800
C	-3.48514400	3.04100600	-2.74701600
H	-4.08269400	3.92506600	-2.53116900
H	-2.73000500	3.25440100	-3.50581200
H	-4.11560900	2.21200800	-3.07519300

Int-24a

C	-1.54745300	-0.31291800	0.75632800
C	-1.88637500	1.02414700	0.22367300
C	-0.97444800	2.05560400	-0.02865000
C	0.39141300	1.87937400	0.13501700
C	0.33846600	-1.27493700	-0.64138300
C	-1.12969600	-1.37862200	-0.42939900
C	1.48480100	2.59835600	-0.56369600
C	2.33154200	1.56465200	-1.47043100
O	0.65679800	-0.10223500	-1.31510600
C	1.98429000	0.14874100	-1.17553900
C	2.56114800	-0.90574400	-0.50532700
C	1.49883100	-1.81431700	-0.16329400
C	-1.78061000	-2.73186800	0.02410100
C	-2.17130100	-2.51295800	1.49868100
C	2.29321300	3.32524600	0.53317000
H	0.73251800	1.10609000	0.81896100
H	-0.67300600	-0.24373800	1.40864100
C	-0.96721800	-3.99656000	-0.23915400
O	-2.99155200	-2.77559900	-0.77348700
H	-1.63803100	-1.07398900	-1.34631200
O	-3.51109400	0.69301800	-1.45172600
C	1.79792700	4.71396600	0.85865700
C	3.32984000	2.75413900	1.15706500
C	3.99290400	-1.00903500	-0.14874100
O	4.79106100	-0.09722700	-0.27232000
O	4.29140100	-2.22000600	0.34094500
C	5.67180200	-2.42093000	0.72205600
H	-1.33058700	2.92518400	-0.57634200
H	1.05936500	3.34905200	-1.23654200
H	2.07753600	1.80102100	-2.50854200
H	3.39757600	1.73172200	-1.32060100
H	1.62566700	-2.72433100	0.40148800
H	-2.93583500	-3.21962200	1.83689900
H	-1.29446400	-2.63198400	2.14766500
H	-1.57823200	-4.87635300	-0.00288800
H	-0.66688800	-4.06031300	-1.28871200
H	-0.07625200	-4.05343300	0.39011400
H	-3.40046300	-3.65035300	-0.65964900
H	1.87317000	5.37226200	-0.01667400
H	0.74447000	4.71414200	1.16806400
H	2.38371200	5.15885800	1.66704200
H	3.87672700	3.29929400	1.92145900
H	3.70511600	1.76451800	0.91385600
H	5.72376700	-3.44793800	1.08062000
H	6.32414500	-2.27682100	-0.14169000
H	5.95155800	-1.71817600	1.51008400
C	-2.67053700	-1.06109600	1.55138200
H	-2.72006500	-0.66836300	2.57102300
C	-3.25546200	1.21540000	-0.38776900
O	-3.95192600	-0.89202300	0.99428500
H	-3.99146000	-1.45760600	0.19437400
O	-3.99987300	2.05757400	0.30654400
C	-5.36103700	2.25082800	-0.16818400
H	-5.35208700	2.58988600	-1.20532800
H	-5.90347200	1.30762500	-0.08153600
H	-5.78523700	3.00655300	0.49007700

Int-24b

C	1.99742900	0.36622700	-0.65762800
C	1.47859400	1.22580300	0.42325900
C	0.16924700	1.73302500	0.43215600
C	-0.68139500	1.74402000	-0.66220800
C	0.20818700	-1.44592400	-0.23090300
C	1.65599200	-1.18919900	-0.37637500
C	-2.16191100	1.77562900	-0.57874000
C	-2.70073400	0.35729500	-1.15051600
O	-0.60365900	-0.72051500	-1.09033300
C	-1.88298800	-0.76099800	-0.62199800
C	-1.92697700	-1.59802900	0.46934900
C	-0.57666500	-2.02834100	0.71703200
C	-2.82030600	2.92619500	-1.34460900
C	-4.32884800	2.92030200	-1.36628800
C	-2.10387800	3.90000000	-1.91566500
H	-0.26382700	1.66947300	-1.66280300
C	2.48238800	-1.90771700	-1.53249500
C	3.63569300	-0.91818300	-1.86057100
C	3.51490600	0.29028000	-0.91564900
H	1.49808500	0.61369200	-1.59898000
H	2.14987600	-1.46521500	0.55692400
O	3.06449000	-3.08416000	-1.00027400
C	1.63491400	-2.23710400	-2.76402400
O	1.98778000	0.39467700	2.55681200
H	3.88246000	1.21778100	-1.37617100
C	-3.07300300	-1.98397600	1.32340400
O	-2.97237600	-2.74365300	2.26174800
O	-4.22323800	-1.39079600	0.93925100
C	-5.38684200	-1.74195100	1.72274900
H	-0.23769900	2.03771100	1.39469500
H	-2.46940900	1.81855200	0.47408400
H	-3.74000900	0.25407000	-0.84149900
H	-2.64828900	0.40027800	-2.24314900
H	-0.26192100	-2.64832500	1.54443700
H	-4.73274700	2.08781600	-1.95517900
H	-4.74131600	2.82836000	-0.35283700
H	-4.70930900	3.84814000	-1.80094800

H	-2.59086200	4.71645400	-2.44041900
H	-1.01852200	3.94404400	-1.87708800
H	3.57234800	-0.58499400	-2.90191400
H	4.59720300	-1.42487000	-1.73842400
H	2.39171800	-3.78466500	-0.97298200
H	1.16059000	-1.34660700	-3.18953800
H	2.27340100	-2.68596700	-3.53123400
H	0.83906100	-2.94945700	-2.51688100
H	-6.21443900	-1.19510400	1.27228400
H	-5.56183400	-2.81873300	1.67404800
H	-5.24509600	-1.44578500	2.76449600
O	4.13685900	0.07319600	0.34140900
H	5.07577500	-0.12549500	0.20058800
C	2.20409300	1.26788100	1.74598100
O	2.96240600	2.33995900	1.88155000
C	3.73241200	2.42533600	3.11280200
H	4.25433700	3.37849600	3.05418900
H	3.06414700	2.39502300	3.97500500
H	4.43633400	1.59224400	3.15362100

TS4a

C	-1.82706900	-0.54721900	0.66675600
C	-1.99627500	0.92159500	0.38360400
C	-0.94316600	1.66636800	-0.01749100
C	0.46230700	1.24124400	0.00634800
C	0.39409100	-0.76553900	-0.47852700
C	-1.02926900	-1.17892200	-0.53129600
C	1.41233900	1.98482300	-0.95852500
C	2.63226400	1.17718800	-1.74288700
O	1.03281100	-0.47557500	-1.70667400
C	2.29621000	-0.19640900	-1.37873300
C	2.65014500	-0.85362700	-0.17212100
C	1.44110100	-1.19931100	0.40181700
C	-1.44282200	-2.70288100	-0.57990700
C	-2.44509200	-2.84769500	0.59152500
C	1.98829900	3.20388800	-0.21273900
H	0.86839000	1.12993800	1.00918700
H	-1.25788500	-0.69503600	1.59770700
C	-0.31039500	-3.72518200	-0.57282200
O	-2.15984300	-2.78320000	-1.83311100
H	-1.43054200	-0.74334000	-1.45076600
O	-3.61647000	2.51851900	-0.37255800
C	1.32598700	4.51996600	-0.53857100
C	2.99130600	3.09264800	0.66328300
C	4.00816700	-0.84806300	0.43596900
O	4.92323300	-0.18050500	0.00063900
O	4.05854000	-1.64890000	1.50269500
C	5.34102200	-1.72186300	2.17851500
H	-1.15669600	2.65840400	-0.41290400
H	0.77986700	2.32558000	-1.78142700
H	2.50637700	1.40201400	-2.80344200
H	3.61656700	1.47554600	-1.39226200
H	1.29592400	-1.57798400	1.40572400
H	-3.18629600	-3.62976800	0.39742200
H	-1.91985900	-3.11166000	1.51795200
H	-0.72282200	-4.73913800	-0.63989900
H	0.35688000	-3.57649200	-1.42796600
H	0.27593700	-3.68476100	0.35116100
H	-2.44293700	-3.70380600	-1.96725200
H	1.41978700	4.75586300	-1.60646400
H	0.25284700	4.51003400	-0.30848200
H	1.78114500	5.33510700	0.03018500
H	3.37855500	3.96428900	1.18199800
H	3.48589700	2.14966300	0.88242000
H	5.19389200	-2.42970800	2.99227400
H	6.10581900	-2.07551200	1.48460400
H	5.61764800	-0.73704500	2.56036400
C	-3.08089500	-1.45630700	0.73011200
H	-3.62435100	-1.32416900	1.66632700
C	-3.33463100	1.61575100	0.38523700
O	-3.98479800	-1.16108300	-0.32034700
H	-3.67678400	-1.62476200	-1.12183500
O	-4.11204300	1.16842800	1.37764100
C	-5.43307700	1.75252200	1.44621800
H	-5.36198300	2.83782800	1.54148400
H	-5.99353700	1.49655100	0.54464900
H	-5.89657300	1.31305400	2.32839000

TS4b

C	2.17232100	-0.20525600	-0.50340800
C	1.91424700	1.05189300	0.28113200
C	0.66241100	1.58208500	0.27120300
C	-0.33113000	1.01463700	-0.61379400
C	-0.29303100	-0.97268900	-0.12449000
C	1.15040000	-1.35387300	-0.13386600
C	-1.75625800	1.51918400	-0.69878900
C	-2.81766600	0.47500700	-1.42109000
O	-1.16346800	-1.11634300	-1.25158400
C	-2.37345800	-0.77271700	-0.78957800
C	-2.46122200	-1.02895700	0.60337100
C	-1.14618800	-1.10634500	1.01746100
C	-1.86904900	2.84430600	-1.46907400
C	-3.19887100	3.54744600	-1.32389600
C	-0.87545000	3.35990700	-2.20131800
H	0.06394700	0.75777400	-1.59517300
C	1.68430800	-2.60101800	-0.93615500
C	3.22460900	-2.33062900	-0.96546500
C	3.49358100	-0.96524900	-0.29985100
H	2.06614500	0.00808300	-1.57778300
H	1.38647800	-1.59780900	0.90514100
O	1.46256600	-3.78779200	-0.18741700
C	1.12137400	-2.75085300	-2.35283400
O	2.64196500	2.24504900	2.22213500
H	4.34152800	-0.43694100	-0.74770400
C	-3.64208200	-0.95753800	1.50707300
O	-3.55572800	-1.10804900	2.70404000
O	-4.76972300	-0.72145800	0.82088000
C	-5.98378500	-0.66642600	1.61369100
H	0.40514900	2.39052200	0.95082900
H	-2.14622500	1.68172900	0.31353800
H	-3.83370600	0.76697800	-1.16573200
H	-2.64787200	0.49686800	-2.49890300
H	-0.80556600	-1.14135700	2.04554400
H	-4.02044900	2.98872000	-1.78925400
H	-3.46139000	3.68938400	-0.26764600
H	-3.16429100	4.53181300	-1.79725300
H	-1.01097000	4.30490100	-2.71883800
H	0.09926200	2.89726200	-2.31090500
H	3.59198100	-2.33186100	-1.99720100
H	3.73541200	-3.13806000	-0.43402500
H	0.62041100	-4.18460600	-0.46041900
H	1.25125600	-1.84090800	-2.94919000
H	1.65090400	-3.56286700	-2.86121500
H	0.05277500	-2.99207600	-2.34400200
H	-6.78485500	-0.49035500	0.89775800
H	-6.12745600	-1.61411100	2.13606600
H	-5.91986800	0.14614500	2.34029500
O	3.65688400	-1.07626600	1.11259200
H	4.45995900	-1.58794300	1.29620300
C	2.92777100	1.72761100	1.16473800
O	4.13776300	1.76245200	0.59062700
C	5.17034600	2.41707900	1.36227100
H	6.06021900	2.39032300	0.73466700
H	4.88127300	3.44633600	1.58436900
H	5.33481300	1.87567400	2.29665500

Int-25a

C	-1.93414200	-0.64558100	0.58219400
C	-2.10661300	0.84791500	0.43564200
C	-1.04002500	1.60006900	0.12320900
C	0.37088500	1.06620100	0.04560000
C	0.40435800	-0.54118700	-0.35618800
C	-0.96613600	-1.12749600	-0.53210600
C	1.25306200	1.94028500	-0.90943100
C	2.67174600	1.25153600	-1.57304400
O	1.13894200	-0.47997500	-1.64642600
C	2.33290300	-0.10175500	-1.22322000
C	2.67467000	-0.78104300	0.01037200
C	1.46617000	-1.08750600	0.55236300
C	-1.21660200	-2.66549300	-0.72120700
C	-2.40403900	-2.95245700	0.23637800
C	1.67362600	3.29052000	-0.37422800
H	0.78730500	1.12852200	1.05707300
H	-1.51301500	-0.87191800	1.57606400
C	-0.02323700	-3.59792700	-0.52831300
O	-1.67576700	-2.74165400	-2.08977200
H	-1.33389800	-0.66882400	-1.45587600
O	-3.63723400	2.67043000	0.16381800
C	1.98725700	4.31891800	-1.43747300
C	1.77646900	3.57677700	0.93135300
C	4.04113000	-0.80732300	0.60299500
O	4.96499900	-0.18717100	0.12123700
O	4.07989700	-1.57194800	1.69315300
C	5.37157100	-1.67254100	2.35050400
H	-1.16623700	2.67309300	0.00348200
H	0.66046200	2.06787400	-1.81924700
H	2.62713700	1.49195600	-2.63532300
H	3.58674000	1.60663300	-1.10361000
H	1.28670000	-1.45626800	1.55559300
H	-3.05374600	-3.74459200	-0.15006700
H	-2.03747200	-3.27547200	1.21861700
H	-0.32977900	-4.63781100	-0.69500600
H	0.77487100	-3.36775200	-1.24133600
H	0.37572200	-3.55305200	0.49151000
H	-1.87406300	-3.67106400	-2.29534600
H	2.80509400	3.99670000	-2.09522000
H	1.11448800	4.50129000	-2.07718500
H	2.28184500	5.27034600	-0.98835200
H	2.08914800	4.56463400	1.25571300
H	1.54949400	2.87343400	1.72596700
H	5.21004400	-2.34352800	3.19212300
H	6.10810600	-2.08275500	1.65717000
H	5.69304300	-0.68610500	2.69018900
C	-3.13398700	-1.60589100	0.37210000
H	-3.82411800	-1.57671600	1.21510800
C	-3.41214500	1.56870400	0.61735000
O	-3.87719500	-1.26335100	-0.78463500
H	-3.43923900	-1.66458900	-1.55706500
O	-4.26522500	0.87092400	1.38237600
C	-5.55436700	1.48747900	1.59523700
H	-5.43289700	2.45975700	2.07761300
H	-6.06765800	1.61571600	0.63994200
H	-6.10073000	0.79923000	2.23893800

Int-25b

C	2.21810300	-0.36718000	-0.46669600
C	2.05885100	0.92530800	0.30528600
C	0.85171700	1.51765500	0.28816200
C	-0.23286900	0.91387200	-0.56439300
C	-0.34518300	-0.66253600	-0.22676500
C	1.02394500	-1.31665300	-0.18462700
C	-1.59068800	1.65476200	-0.58482500
C	-2.80730000	0.80116100	-1.38822300
O	-1.29847400	-0.95440500	-1.38307000
C	-2.41755100	-0.49481800	-0.86666600
C	-2.54080800	-0.87848700	0.52686600
C	-1.24872700	-0.93659500	0.93191700
C	-1.56764200	3.03659700	-1.21789900
C	-2.72522200	3.93324400	-0.84278700
C	-0.59493000	3.45047800	-2.04041600
H	0.13173500	0.87674600	-1.59877900
C	1.36285700	-2.62504000	-0.94304200
C	2.92837100	-2.66357200	-0.78631800
C	3.38204000	-1.32392700	-0.14491800
H	2.26207900	-0.13861000	-1.54334700
H	1.15588300	-1.60356800	0.86434900
O	0.69250100	-3.65425800	-0.21822200
C	0.96043000	-2.67122800	-2.42193800
O	2.95894000	2.36161700	1.99483100
H	4.33188100	-0.96427000	-0.55147000
C	-3.74867100	-0.92756300	1.40000800
O	-3.69822200	-1.23208200	2.56752700
O	-4.84883400	-0.60053400	0.70820200
C	-6.09357400	-0.63842900	1.45579900
H	0.67317800	2.43580900	0.83919800
H	-1.97191300	1.74645000	0.43846700
H	-3.77880600	1.16765700	-1.06527000
H	-2.64283500	0.91049500	-2.45978700
H	-0.90674100	-1.03458500	1.95675900
H	-3.68911500	3.53046800	-1.17989400
H	-2.79269700	4.05856900	0.24557200
H	-2.61146900	4.92384900	-1.28948600
H	-0.61469900	4.45437300	-2.45398500
H	0.25645200	2.84128700	-2.32406700
H	3.41116600	-2.80777000	-1.75894200
H	3.20889100	-3.50465800	-0.14524300
H	0.80873700	-4.49376800	-0.69212600
H	1.36335500	-1.82299300	-2.98695700
H	1.36980300	-3.58027500	-2.88014000
H	-0.12463300	-2.68985100	-2.54060000
H	-6.86556900	-0.36527200	0.73857000
H	-6.25860900	-1.64459600	1.84539700
H	-6.05361400	0.07409300	2.28208000
O	3.44019500	-1.39964000	1.27793900
H	4.18274900	-1.97022200	1.52932200
C	3.15596700	1.61276800	1.06245700
O	4.36472600	1.35693400	0.53337800
C	5.47430700	2.00942200	1.18774500
H	6.35772500	1.71589500	0.62161300
H	5.34237500	3.09336300	1.16662300
H	5.54748400	1.67487800	2.22510400

Int-26a

C	-2.08617700	-0.89718400	0.55741500
C	-2.51695400	0.54871400	0.49832400
C	-1.57964400	1.49225800	0.31964200
C	-0.09561400	1.22582100	0.28786300
C	0.29475300	-0.28472200	-0.11779500
C	-0.93901600	-1.10085200	-0.45996600
C	0.70005500	2.20688800	-0.62776000
C	2.20777600	1.82677800	-0.68616700
O	1.16210000	-0.25230900	-1.28131300
C	2.37514100	0.29091100	-0.80127200
C	2.49453500	-0.38714900	0.57670500
C	1.25151300	-0.72919600	0.96817500
C	-0.88071700	-2.63816700	-0.77186700
C	-2.08408300	-3.21214600	0.02326000
C	0.50482500	3.67393900	-0.23948400
H	0.26017200	1.40176400	1.31579100
H	-1.73385700	-1.13107900	1.57695700
C	0.43489200	-3.36611200	-0.50464000
O	-1.17966400	-2.69254800	-2.18753100

H	-1.28615000	-0.64745100	-1.39419900
O	-4.33064900	2.10172100	0.28715700
C	-0.13410700	4.55194000	-1.28623800
C	0.88382800	4.14543000	0.95305800
C	3.75168300	-0.60921100	1.22225300
O	4.86352600	-0.45159000	0.57410700
O	3.79645200	-0.98815100	2.45546800
C	5.09163100	-1.28802000	3.07645600
H	-1.88741500	2.53381900	0.28725000
H	0.30342400	2.06890400	-1.63865200
H	2.65800100	2.32192900	-1.55220700
H	2.73994200	2.20015800	0.19525800
H	0.95895100	-1.16497200	1.91713900
H	-2.53247500	-4.07427100	-0.48228400
H	-1.76367300	-3.54349600	1.01908000
H	0.33691200	-4.42623600	-0.76995700
H	1.24370800	-2.93596500	-1.10124600
H	0.71052300	-3.33547200	0.55638900
H	-1.22459800	-3.62751900	-2.45028100
H	0.44917700	4.54664600	-2.21721500
H	-1.13853900	4.19149700	-1.54580700
H	-0.22205800	5.58744500	-0.94667100
H	0.74575800	5.18865000	1.22148300
H	1.34137000	3.51691700	1.71485600
H	4.83731400	-1.61740600	4.08089600
H	5.58937600	-2.07636400	2.51063300
H	5.69742600	-0.38147500	3.09660400
C	-3.06246400	-2.03326000	0.15928200
H	-3.82520500	-2.19684000	0.92021500
C	-3.93315000	1.01319900	0.64713100
O	-3.74640000	-1.74763300	-1.04959700
H	-3.14802400	-1.95879700	-1.78891500
O	-4.69970600	0.10070400	1.27229900
C	-6.08413900	0.46850200	1.43926000
H	-6.16516400	1.38528100	2.02781700
H	-6.55164900	0.62202400	0.46411700
H	-6.54592200	-0.36934400	1.96089000
C	3.46160400	0.13343900	-2.99189900
H	2.53616600	-0.25201100	-3.42347000
H	4.31842900	-0.39964700	-3.40364600
H	3.56246200	1.20426000	-3.19017600
O	3.47909600	-0.13944600	-1.57227400
H	4.64054900	-0.26773500	-0.39761000

Int-26b

C	2.36630800	-0.57816600	-0.49841200
C	2.48253600	0.73939200	0.23538100
C	1.40026200	1.53568500	0.25642400
C	0.16921800	1.12812700	-0.50400000
C	-0.22802800	-0.37509700	-0.21012900
C	1.02372100	-1.27386800	-0.14989500
C	-1.05239700	2.06439600	-0.37310700
C	-2.29385200	1.42820400	-1.07832500
O	-1.20419100	-0.70445700	-1.23718700
C	-2.39795300	-0.08136800	-0.79250000
C	-2.36099100	-0.36823800	0.71116700
C	-1.06351900	-0.47549300	1.05069000
C	-0.83236400	3.48564300	-0.88675300
C	-1.76122000	4.52781100	-0.30828300
C	0.09956400	3.80652300	-1.78980500
H	0.42966900	1.06522000	-1.57115100
C	1.10005500	-2.65375700	-0.84634700
C	2.63319400	-2.98079800	-0.71353200
C	3.34817200	-1.71701400	-0.16675400
H	2.41727200	-0.39635000	-1.58349800
H	1.14138400	-1.53151300	0.90779400
O	0.27465800	-3.52121100	-0.06192500
C	0.66854700	-2.70854000	-2.31729300
O	3.73181600	2.02310500	1.82664100
H	4.32879000	-1.55781400	-0.62514800
C	-3.53683600	-0.50570300	1.51478400
O	-4.70562200	-0.59845700	0.96099700
O	-3.44639800	-0.56344700	2.79996600
C	-4.64922000	-0.80234100	3.60427600
H	1.43028200	2.48970300	0.77402300
H	-1.29039700	2.15214500	0.69725500
H	-3.21384200	1.93846000	-0.76963100
H	-2.18629000	1.55977600	-2.15983700
H	-0.64933400	-0.59817400	2.04524700
H	-2.81764500	4.28054900	-0.48009500
H	-1.62926200	4.61157900	0.77915800
H	-1.57732800	5.51250400	-0.74571700
H	0.20859600	4.82914200	-2.13964400
H	0.79562900	3.08745800	-2.21015600
H	3.05270900	-3.27260900	-1.68272200
H	2.76939600	-3.81972700	-0.02402100
H	0.25128900	-4.38862800	-0.49733900
H	1.19899000	-1.96815400	-2.92692100
H	0.91415400	-3.69562300	-2.73043400
H	-0.40373800	-2.54269300	-2.41829200
H	-4.28254300	-0.84959000	4.62669800
H	-5.34241200	0.02845200	3.46801400
H	-5.10545500	-1.74472500	3.29902100
O	3.46273500	-1.74011400	1.25709200
H	4.11666700	-2.41327400	1.49981100
C	3.72513400	1.23448900	0.90501800
O	4.83950700	0.75902200	0.31340600
C	6.08032200	1.21931500	0.88344300
H	6.86488100	0.76392300	0.27926800
H	6.14013900	2.30909900	0.83678300
H	6.15897700	0.90057000	1.92575400
C	-3.75314100	-0.61780300	-2.76070600
H	-2.83498600	-0.88948900	-3.28535000
H	-4.07806600	0.38654400	-3.04651200
H	-4.53674500	-1.34039200	-2.98894300
O	-3.54040900	-0.71062600	-1.33420400
H	-4.57262400	-0.64528200	-0.04375700

1

C	2.35270600	-0.50883300	-0.47366100
C	2.39455400	0.83259600	0.22667400
C	1.26416800	1.56193500	0.21959300
C	0.07026000	1.05713200	-0.53949300
C	-0.25307200	-0.45286000	-0.19641200
C	1.05308000	-1.27180200	-0.09495100
C	-1.20116700	1.93145000	-0.46402100
C	-2.39586000	1.21278500	-1.16954700
O	-1.17067900	-0.85529600	-1.22956800
C	-2.43885200	-0.29150600	-0.82369400
C	-2.40554000	-0.48969900	0.68829200
C	-1.12137000	-0.56058300	1.04740900
C	-1.04314900	3.34530000	-1.01366700
C	-2.07625700	4.33825700	-0.53203700
C	-0.08362600	3.71325800	-1.87000700
H	0.35795100	0.98378600	-1.59964500
C	1.21990000	-2.67013800	-0.74227600
C	2.77889100	-2.87786300	-0.67806000
C	3.41543900	-1.57107800	-0.14839300
H	2.37581600	-0.34773100	-1.56408200
H	1.18057300	-1.48039800	0.97224100
O	0.52357200	-3.58724500	0.10928600
C	0.72364200	-2.82264900	-2.18518700
O	3.58291300	2.23851300	1.76930200
H	4.37680800	-1.35147100	-0.62632500
C	-3.59182700	-0.35324600	1.55251300
O	-4.61436200	0.21998100	1.23015600

O	-3.39748000	-0.91481800	2.76847500
C	-4.49422300	-0.77678000	3.68492600
H	1.23459800	2.52871000	0.71387800
H	-1.46700900	2.03569200	0.59717100
H	-3.34696500	1.67098800	-0.88111600
H	-2.27474600	1.32011000	-2.25275400
H	-0.72871300	-0.60609200	2.05645600
H	-3.09676700	4.01158400	-0.76964900
H	-2.03163700	4.45048100	0.55993600
H	-1.92526500	5.32436600	-0.98124600
H	-0.02879100	4.73225700	-2.24474100
H	0.68416600	3.03411900	-2.22668300
H	3.18089200	-3.11924100	-1.66953200
H	3.00659000	-3.71960500	-0.01521200
H	0.41013200	-4.41764900	-0.37919400
H	1.15358800	-2.06022900	-2.84478300
H	1.03700900	-3.80128700	-2.57619000
H	-0.36184300	-2.74385900	-2.22991200
H	-4.17898300	-1.28763900	4.59523000
H	-4.69882200	0.27836100	3.88723200
H	-5.39623500	-1.23999600	3.27630200
O	3.56853000	-1.57998900	1.27349800
H	4.15036400	-2.31949400	1.50530400
C	3.60079400	1.41787900	0.87377200
O	4.74895600	0.98543200	0.29561800
C	5.95227700	1.51209300	0.87117400
H	6.76886200	1.08078100	0.29041100
H	5.96811500	2.60321700	0.80422400
H	6.03299300	1.22122300	1.92220500
C	-3.62502400	-0.93343400	-2.80373400
H	-2.67692900	-1.13092400	-3.31735500
H	-4.01875100	0.03946800	-3.12460800
H	-4.34672700	-1.70844300	-3.07150300
O	-3.48079000	-1.01087500	-1.38943300

2

C	-2.05007000	-0.83413900	0.55388100
C	-2.45678900	0.61964100	0.47958000
C	-1.49116500	1.53464900	0.28433600
C	-0.01925600	1.20484600	0.26858600
C	0.33015300	-0.30167100	-0.16297900
C	-0.93997200	-1.06677800	-0.50196100
C	0.82690500	2.18531500	-0.59938900
C	2.31955600	1.74610000	-0.67485300
O	1.15731300	-0.25618500	-1.33723500
C	2.43550400	0.20675100	-0.86328000
C	2.54088500	-0.50710500	0.48932000
C	1.30455100	-0.82871500	0.87954900
C	-0.95025000	-2.60039100	-0.82670000
C	-2.08793100	-3.15533600	0.06974700
C	0.70278000	3.64516600	-0.16409100
H	0.31677200	1.32699300	1.30939300
H	-1.64686300	-1.04513600	1.55913900
C	0.36397400	-3.36684700	-0.71771200
O	-1.40525500	-2.63871800	-2.21022400
H	-1.29039500	-0.59205500	-1.42452800
O	-4.26601400	2.18179500	0.23443900
C	0.27001500	4.61376300	-1.23713600
C	0.98001200	4.05134100	1.07942900
C	3.79231500	-0.59505700	1.26411600
O	4.76570300	0.10782800	1.08163700
O	3.71880100	-1.54006900	2.23335400
C	4.88707600	-1.65057700	3.06200700
H	-1.76859000	2.58432100	0.23029900
H	0.42768700	2.10478000	-1.61643900
H	2.79694800	2.26975100	-1.50958000
H	2.86655800	2.04281600	0.22458100
H	1.01969700	-1.25583700	1.83439000
H	-2.57228800	-4.03178100	-0.37620800
H	-1.69023900	-3.45582800	1.04768200
H	0.21142600	-4.41136800	-1.02247600
H	1.12462500	-2.91848300	-1.36078600
H	0.74315400	-3.38221100	0.30867500
H	-1.45403400	-3.57288700	-2.47393100
H	0.94878900	4.57440600	-2.10043200
H	-0.72950900	4.36025600	-1.61626800
H	0.24525100	5.64470200	-0.87095700
H	0.90224200	5.09665500	1.36656700
H	1.29937000	3.36422100	1.85967400
H	4.66809700	-2.45072400	3.76979700
H	5.76338600	-1.90020600	2.45807700
H	5.07687600	-0.71165000	3.58951600
C	-3.05378100	-1.96845100	0.24336500
H	-3.76922300	-2.11535000	1.05350600
C	-3.85587800	1.10867300	0.63130700
O	-3.81793900	-1.71893400	-0.92851700
H	-3.23361900	-1.89729200	-1.68947500
O	-4.62627800	0.23407800	1.31963900
C	-5.99792000	0.62452200	1.47927200
H	-6.06892300	1.57140800	2.02128600
H	-6.47796600	0.73539800	0.50355400
H	-6.46701800	-0.17988200	2.04696000
C	3.31949600	0.10149000	-3.08390800
H	2.39709700	-0.31090200	-3.50622900
H	4.18287700	-0.34757200	-3.57933400
H	3.34147800	1.18600900	-3.25465300
O	3.45055500	-0.23050800	-1.70518300

H_2O

O	0.00000000	0.00000000	0.11971800
H	0.00000000	0.76155000	-0.47887400
H	0.00000000	-0.76155000	-0.47887400

H_3O^+

H	0.00000000	0.94571000	-0.20855900
H	0.81900900	-0.47285500	-0.20855900
H	-0.81900900	-0.47285500	-0.20855900
O	0.00000000	0.00000000	0.07821000

CH_3OH

C	0.66231900	-0.01953800	0.00000000
H	1.07973500	0.99102700	-0.00000100
H	1.03696900	-0.54365000	-0.89314900
H	1.03696900	-0.54364900	0.89315000
O	-0.74918300	0.12249500	0.00000000
H	-1.13412600	-0.76646000	0.00000000
